# Pressure-induced postsynthetic cluster anion substitution in a MIL-53 topology scandium metal–organic framework†

**DOI:** 10.1039/d3sc00904a

**Published:** 2023-06-19

**Authors:** Alexander J. R. Thom, Gemma F. Turner, Zachary H. Davis, Martin R. Ward, Ignas Pakamorė, Claire L. Hobday, David R. Allan, Mark R. Warren, Wai L. W. Leung, Iain D. H. Oswald, Russell E. Morris, Stephen A. Moggach, Sharon E. Ashbrook, Ross S. Forgan

**Affiliations:** a WestCHEM School of Chemistry, University of Glasgow Joseph Black Building, University Avenue Glasgow G12 8QQ UK ross.forgan@glasgow.ac.uk; b School of Molecular Sciences, The University of Western Australia 35 Stirling Highway, Crawley Perth Western Australia 6009 Australia stephen.moggach@uwa.edu.au; c EaStCHEM School of Chemistry and Centre of Magnetic Resonance, University of St Andrews St Andrews KY16 9ST UK sema@st-andrews.ac.uk; d Strathclyde Institute of Pharmacy & Biomedical Sciences (SIPBS), University of Strathclyde 161 Cathedral Street Glasgow G4 0RE UK; e EaStCHEM School of Chemistry and Centre for Science at Extreme Conditions, The University of Edinburgh King's Buildings, David Brewster Road Edinburgh EH9 3FJ UK; f Diamond Light Source Ltd, Harwell Science and Innovation Campus Didcot Oxfordshire OX11 0DE UK

## Abstract

Postsynthetic modification of metal–organic frameworks (MOFs) has proven to be a hugely powerful tool to tune physical properties and introduce functionality, by exploiting reactive sites on both the MOF linkers and their inorganic secondary building units (SBUs), and so has facilitated a wide range of applications. Studies into the reactivity of MOF SBUs have focussed solely on removal of neutral coordinating solvents, or direct exchange of linkers such as carboxylates, despite the prevalence of ancillary charge-balancing oxide and hydroxide ligands found in many SBUs. Herein, we show that the μ_2_-OH ligands in the MIL-53 topology Sc MOF, GUF-1, are labile, and can be substituted for μ_2_-OCH_3_ units through reaction with pore-bound methanol molecules in a very rare example of pressure-induced postsynthetic modification. Using comprehensive solid-state NMR spectroscopic analysis, we show an order of magnitude increase in this cluster anion substitution process after exposing bulk samples suspended in methanol to a pressure of 0.8 GPa in a large volume press. Additionally, single crystals compressed in diamond anvil cells with methanol as the pressure-transmitting medium have enabled full structural characterisation of the process across a range of pressures, leading to a quantitative single-crystal to single-crystal conversion at 4.98 GPa. This unexpected SBU reactivity – in this case chemisorption of methanol – has implications across a range of MOF chemistry, from activation of small molecules for heterogeneous catalysis to chemical stability, and we expect cluster anion substitution to be developed into a highly convenient novel method for modifying the internal pore surface and chemistry of a range of porous materials.

## Abstract 2

Application of pressure induces reactivity of pore-bound methanol with the secondary building unit of the metal–organic framework GUF-1(Sc), substituting coordinated bridging hydroxide ligands for bridging methoxides, in single crystals and in bulk.

## Graphical abstract

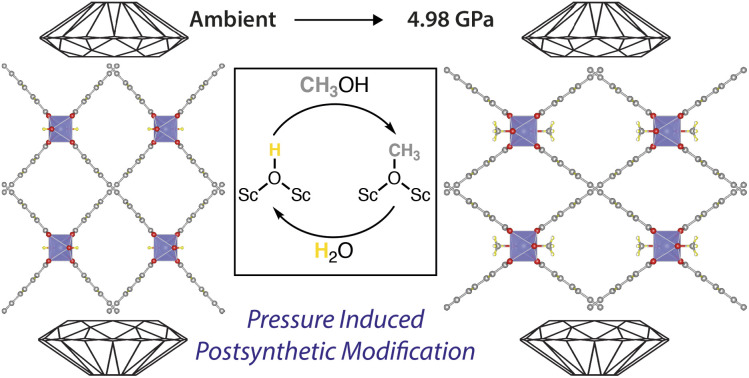

## Introduction

Metal–organic frameworks (MOFs), coordination polymers wherein metal ions/clusters are connected by multitopic linkers into network structures,^1^ are being extensively investigated for a number of applications based around the chemistry of the inorganic secondary building unit (SBU). Kinetically labile bonds between metals and ligands can allow access to coordinatively unsaturated metal sites^2–4^ that endow MOFs with catalytic properties,^5–7^ enhance their interactions with sorbates such as H_2_,^8–11^ and offer potential sensing mechanisms.^12–14^ Dynamic SBU solvent substitution can facilitate structural flexibility^15,16^ and metal–ion exchange,^17,18^ while also offering routes to pore functionalisation through linker exchange,^18–22^ linker incorporation/grafting,^23–26^ and defect substitution.^27–33^ Coordinative exchange reactions may also be responsible for MOF degradation, for example through hydrolysis.^34^ To date, most studies on such reactions within MOFs focus either on binding and removal of neutral solvent molecules,^35^ or direct exchange of carboxylate and/or pyridyl-based ligands, despite the fact that a significant number of commonly observed SBUs contain bridging oxo or hydroxo ligands.^36^ Some of us have recently developed solid-state (and in particular ^17^O) NMR spectroscopy as a valuable analytical tool to study structural and chemical changes in microporous materials such as MOFs and zeolites.^37–40^ In doing so, we have shown that the bridging μ_2_-OH ligand in the one-dimensional chain SBU of MIL-53(Sc), [ScOH(BDC)]_*n*_ (BDC = 1,4-benzenedicarboxylate), is labile, observing up to 25% enrichment with ^17^O by reaction with H_2_^17^O (90% ^17^O) under hydrothermal conditions (200 °C, 72 h).^38^ Allied to the fact that MIL-53 analogues are known with alternative anionic^41–43^ and neutral^44–46^ bridging ligands, this suggests a rich potential reactivity at this particular SBU.

Herein, we report the pressure-induced reactivity of the SBU of the Sc MOF GUF-1 (GUF = Glasgow University Framework), where the 4,4′-(ethyne-1,2-diyl)dibenzoate (EDB^2−^) linker connects one-dimensional ScOH SBUs into a two-fold interpenetrated MIL-53 structure with **sra** topology and overall formula [ScOH(EDB)]_*n*_.^47^ Using high-pressure techniques alongside solid-state NMR spectroscopy, we show that up to 17(2)% of bridging μ_2_-OH ligands can be replaced by bridging μ_2_-OCH_3_ units in the bulk phase by reaction with methanol at 0.8 GPa in a large volume press, with conversions of up to 98(4)% in a single crystal pressurised to 4.98 GPa in methanol in a diamond anvil cell (DAC) observed by single crystal X-ray diffraction. This is at least an order of magnitude higher than analogous reactivity under ambient conditions, which we hypothesise is a consequence of the pressure-induced confinement of the CH_3_OH within the pores of the MOF inducing enhanced reactivity in a confined space,^48^ and represents a rare example of pressure-induced postsynthetic modification of MOFs^49^ with significant implications for their activation with methanol, and processing and shaping for application.

## Results and discussion

**Fig. 1:**
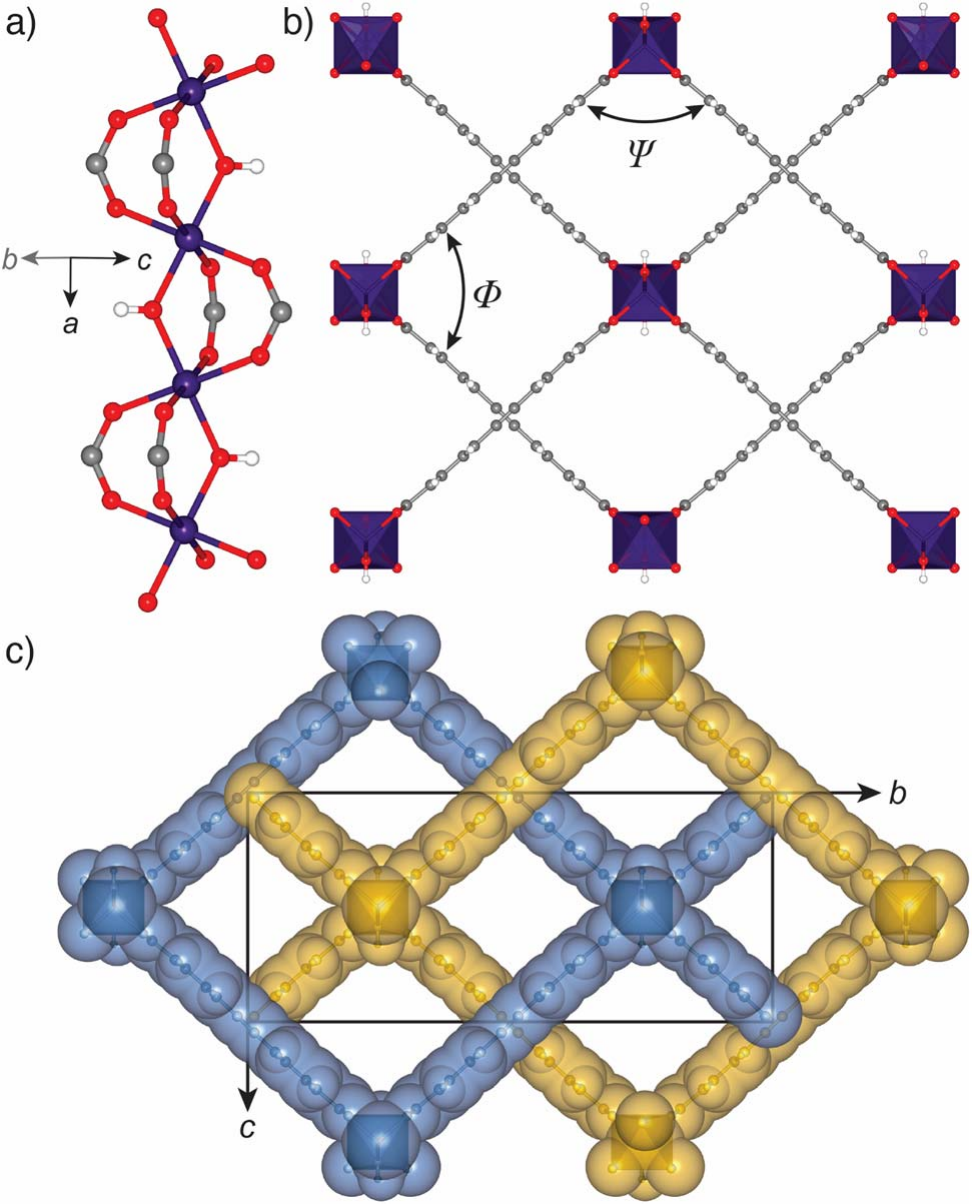
Solid state-structure of GUF-1-(HCl). (a) Infinite chain SBU of ScO_6_ octahedra with bridging μ_2_-OH ligands that runs down the crystallographic *a*-axis. (b) Fragment of the packing structure viewed down the crystallographic *a*-axis, with interior pore angles *Ψ* and *Φ* labelled. (c) Two-fold interpenetrated packing structure with individual nets coloured blue and yellow, and the unit cell overlaid. Where applicable, atoms are coloured Sc: purple; C: grey; O: red; H: white. H atoms and pore-bound DMF in parts (b) and (c) are omitted for clarity. Reproduced from CSD deposition 2095589.^47^

We recently reported the synthesis, structure, and excellent H_2_ storage capacity of GUF-1.^47^ As its interpenetration results in limited breathing in comparison to non-interpenetrated MIL-53 topology MOFs,^50,51^ allied to the fact that it has a potentially flexible EDB^2−^ linker, we sought to characterise its structural response to pressure using high-pressure single crystal X-ray diffraction in a DAC. This approach^52^ has previously allowed us and others to characterise mechanical compliance of a range of MOFs,^53–58^ as well as to investigate the structural basis of spectroscopic responses to pressure and different guests,^59,60^ complementing a burgeoning body of work investigating pressure compliance of MOFs.^61–69^ Single crystals of GUF-1-(HCl) were prepared according to our previously reported HCl modulated solvothermal synthesis in *N*,*N*-dimethylformamide (DMF),^47^ and transferred into fresh DMF for storage (see ESI, Section S2†). Under ambient conditions, GUF-1 forms pale pink cuboidal crystals in the orthorhombic space group, *Cmme*. One-dimensional chains of corner-sharing ScO_6_ octahedra extend along the crystallographic *a*-axis (Fig. 1a). The chains are tethered at the Sc^iii^ centres by bridging EDB^2−^ linkers to form a wine-rack net in the *bc* plane of the unit cell (Fig. 1b), which is two-fold interpenetrated and perforated by rhombic, one-dimensional channels that run parallel to the ScO_6_ chains down the *a*-axis (Fig. 1c). We have previously quantified the flexibility of the material by measuring the internal angles of the rhombic pore, *Ψ* and *Φ* (Fig. 1b). There are two types of chemically distinct channels. One is decorated by μ_2_-OH groups at the shared corners of ScO_6_ pairs, and contains two DMF molecules from the crystallisation solvent per unit formula, with a hydrogen bonding interaction between the formyl group of the DMF and the H atom of the bridging hydroxide (O⋯O = 2.89(2) Å).^47^ The second channel is vacant upon synthesis, with a solvent accessible volume of 270 Å^3^ (probe radius = 1.2 Å, grid spacing = 0.7 Å, Mercury, CSD).^70^

**Fig. 2:**
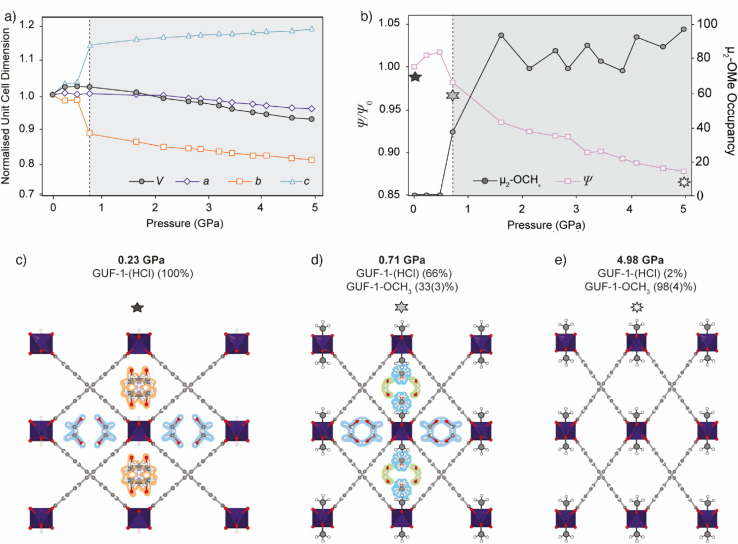
(a) Unit cell volume (black) and normalised unit cell axis lengths (*a* – purple diamonds, *b* – orange squares, *c* – blue triangles) of GUF-1-(HCl) (white region) and GUF-1-OCH_3_ (shaded region) during hydrostatic compression in CH_3_OH. Error bars are within the data markers. (b) Plot of the refined occupancy of the exchanged μ_2_-OCH_3_ bridge (grey circles) and the normalised hinge angle, *Ψ*/*Ψ*_0_ (pink squares), where *Ψ* is at high-pressure and *Ψ*_0_ is at ambient pressure, during compression. The stars correspond to individual structures shown in (c)–(e). (c) Crystal structure of GUF-1-(HCl) at 0.23 GPa viewed along the crystallographic *a*-axis, showing disordered DMF (highlighted in orange) and CH_3_OH (highlighted in blue) in the pores. (d) Crystal structure of the post-synthetically modified framework, GUF-1-OCH_3_, at 0.71 GPa with the CH_3_ groups enhanced for clarity. Adsorbed water in the pores is highlighted in green. At this pressure, 33(3)% of the μ_2_-OH groups have been exchanged for μ_2_-OCH_3_. (e) Structure of GUF-1-OCH_3_ at 4.98 GPa, where 98(4)% of the μ_2_-OH groups have been exchanged for μ_2_-OCH_3_ and the wine-rack structure is compressed. Atoms are coloured according to previous figure.

A single crystal of GUF-1-(HCl) was transferred from DMF storage and compressed in a modified Merrill–Bassett DAC^71^ using methanol (CH_3_OH) as the pressure-transmitting medium; CH_3_OH is a commonly used solvent which is small enough to infiltrate the framework pores and remains liquid to high pressures (see ESI, Section S3†). Compression was followed by *in situ* single crystal X-ray diffraction up to 4.98 GPa. A crystal of GUF-1-(HCl) was also compressed in a non-penetrating medium of Fluorinert® FC-70 to examine its flexibility under direct pressure. However, the crystal became polycrystalline at the initial loading pressure of 0.1 GPa, so no further structural analysis was performed. Crystallographic and structural data are given in Tables 1, S2 and S3 (ESI†). Compression of GUF-1-(HCl) in CH_3_OH between ambient pressure and 4.98 GPa causes the unit cell volume to decrease by 158.9(9) Å (−7.0%) (Fig. 2a, Table 1).

**Table 1:** Unit cell axes and refined crystallographic occupancy of μ_2_-OCH_3_ for GUF-1 and GUF-1-OCH_3_ during hydrostatic compression in a pressure transmitting medium of CH_3_OH. All structures are in the space group *Cmme*

*P*/GPa	*a*/Å	*b*/Å	*c*/Å	*V*/Å^3^	μ_2_-OCH_3_ occ.
0.00^a^	7.3054 (5)	26.5207 (17)	11.7550 (9)	2277.5 (5)	0.00
0.23	7.3533 (9)	26.584 (12)	11.879 (4)	2322.2 (14)	0.00
0.47	7.3205 (15)	26.609 (5)	11.922 (2)	2322.2 (8)	0.00
0.71	7.3445 (19)	23.984 (3)	13.1770 (13)	2321.1 (7)	0.33 (3)
1.61	7.3293 (16)	23.332 (3)	13.3614 (11)	2284.9 (6)	0.93 (4)
2.13	7.290 (3)	22.957 (4)	13.4286 (14)	2247.4 (9)	0.69 (5)
2.61	7.241 (4)	22.814 (7)	13.473 (3)	2225.6 (14)	0.82 (5)
2.84	7.213 (2)	22.789 (3)	13.4966 (13)	2218.6 (7)	0.72 (4)
3.20	7.180 (2)	22.587 (4)	13.5541 (13)	2198.2 (7)	0.85 (4)
3.45	7.1429 (17)	22.463 (5)	13.5504 (12)	2174.2 (7)	0.78 (5)
3.85	7.1069 (17)	22.297 (4)	13.5859 (16)	2152.8 (7)	0.68 (4)
4.11	7.0769 (18)	22.232 (6)	13.613 (15)	2141.7 (6)	0.90 (4)
4.60	7.0380 (14)	22.061 (7)	13.652 (2)	2119.8 (8)	0.85 (4)
4.98	7.0142 (12)	21.941 (7)	13.6972 (19)	2108.0 (8)	0.98 (4)

a Separate crystal, data collection at ambient pressure at 273 K.

The compression occurs in two stages, marked by a change in the compressibility of the crystal at 0.71 GPa. Initial compression of native GUF-1 between ambient pressure and 0.47 GPa causes the unit cell volume to increase by 55.3(9) Å^3^ (+2.4%) with the associated change in the channel shape indicated by an increase in *Ψ*, the hinge angle of the framework defined as the angle between intersecting EDB^2−^ linkers^47^ (Fig. 2b, Table 1). Compression of native GUF-1 promotes adsorption of CH_3_OH into the previously vacant channels at 0.23 GPa, expanding the structure (Fig. 2c). Subsequently, postsynthetic modification occurs suddenly at a critical pressure of 0.71 GPa, (Fig. 2d) and involves partial exchange of the μ_2_-OH ligand for a bridging methoxide group, μ_2_-OCH_3_, in a single-crystal-to-single-crystal reaction with retention of the *Cmme* symmetry. The high-pressure phase observed between 0.71 GPa and 4.98 GPa (Fig. 2e) therefore corresponds to a postsynthetically modified framework, denoted as GUF-1-OCH_3_. At 0.71 GPa, conversion from GUF-1 to GUF-1-OCH_3_ is measured to be 33(3)%, according to the refined crystallographic occupancy of the C atom of the methyl group.

Postsynthetic cluster anion substitution is clearly facilitated by the pressure-induced intrusion of CH_3_OH from the hydrostatic medium into the framework channels. At 0.71 GPa, the pressure is sufficient to promote exchange of the DMF in the μ_2_-OH decorated channel for CH_3_OH and H_2_O (Fig. 2d), which brings the reactant CH_3_OH in close proximity to the μ_2_-OH sites, (O⋯O = 5.2(3) Å), likely promoting the postsynthetic cluster anion exchange. The subsequent identification of adsorbed H_2_O could result from this being the by-product of the μ_2_-OH for μ_2_-OCH_3_ exchange or, alternatively, it may originate from residual moisture in the pressure transmitting medium. The CH_3_OH is adsorbed at two independent sites near the centre of the channel, with occupancies of ∼20% at 0.71 GPa, one of which is disordered over a mirror plane, while H_2_O is located in two equivalent sites near the corner of the rhombic channel, with an occupancy of ∼49%. Above 0.71 GPa, the adsorbate became highly disordered and so was modelled using the SQUEEZE^72^ algorithm in PLATON.^73^

The mechanism of the postsynthetic cluster anion substitution process cannot be ascertained from the crystal structure, although it is possible that the guest exchange and cluster anion exchange are concerted, accounting for the high pressure required to facilitate the reaction. Intuitively, μ_2_-OH to μ_2_-OCH_3_ exchange is likely to be an associative process involving coordination of methanol and breaking of one of the Sc–OH bonds, maintaining the overall six-coordinate environment around the Sc^iii^ ions, followed by proton transfer from methanol to hydroxide, water dissociation, and subsequent coordination to the now bridging μ_2_-OCH_3_ unit. Confinement of H_2_O in the channel may promote the reverse process, which would account for the partial conversion of GUF-1 to GUF-1-OCH_3_ at 0.71 GPa.

Conversion is effectively quantitative by 1.61 GPa, being measured as 93(4)%. The possible equilibrium between μ_2_-OH, μ_2_-OCH_3_, H_2_O and CH_3_OH may create a pressure dependence on the conversion to GUF-1-OCH_3_; values fluctuate between 1.61 GPa and 4.98 GPa before reaching 98(4)% at the highest pressure measured, with pressure-induced intrusion of CH_3_OH into the channels favouring the forward, μ_2_-OH to μ_2_-OCH_3_, exchange process (Fig. 2b, Table 1). While we appreciate the refined C-atom occupancy for the μ_2_-OCH_3_ unit does fluctuate above 1.61 GPa, this is a room temperature measurement from a single crystal in a DAC. Nevertheless, the largest and most statistically significant change occurs on increasing the pressure from 0.71 to 1.61 GPa (12*σ* change in C-atom occupancy), after which the refined occupancies all lie within 0.8–3.9*σ* of each other.

**Fig. 3:**
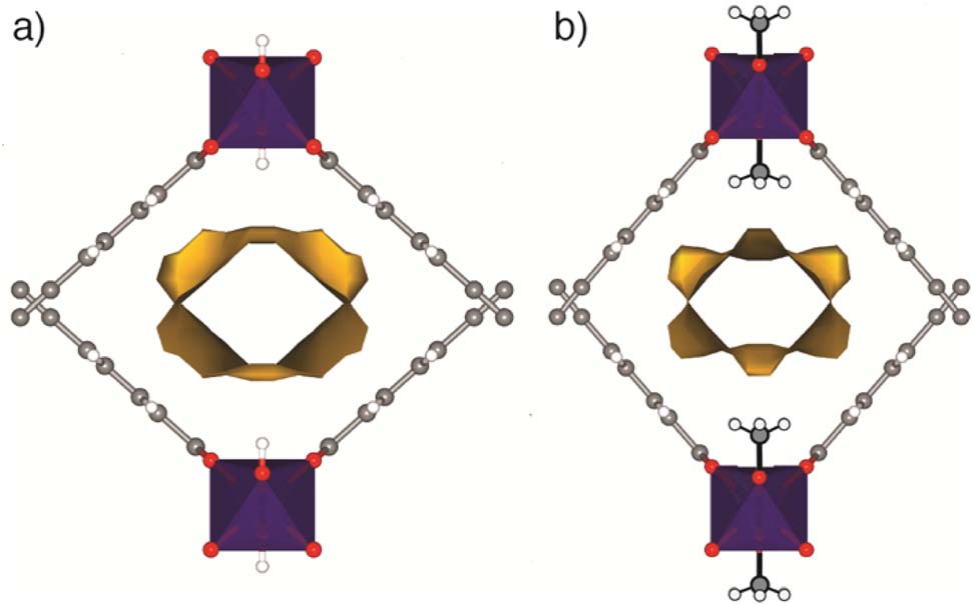
Fragments of (a) GUF-1-(HCl) (ambient pressure) and (b) GUF-1-OCH_3_ (0.71 GPa) showing the solvent accessible volume (yellow).

The μ_2_-OH to μ_2_-OCH_3_ substitution process is associated with a decrease in the porosity of the framework, and occurs concurrently with compression of the wine-rack structure. In GUF-1-OCH_3_, the methyl groups of the μ_2_-OCH_3_ ligands protrude into the channel, decreasing the pore volume by 39 Å^3^ (−13.1%) between 0.47 GPa and 0.71 GPa (Fig. 3 and Table S3†), and increasing its hydrophobicity.

At the time of formation of GUF-1-OCH_3_, the hinge angle, *Ψ*, decreases from 97.41(3)° to 94.30(2)°, compressing the wine-rack structure in width and extending it in height (Table S3†). This is associated with a sudden contraction of the *b*-axis by 2.625(6) Å (−9.9%) and extension of the *c*-axis by 1.255(2) Å (+10.5%) between 0.47 GPa and 0.71 GPa, while the *a*-axis and cell volume remain largely unchanged (Table 1). This type of anisotropic compression and associated negative linear compressibility are common to MIL-53 frameworks^74–77^ and, in GUF-1-OCH_3_, appears to primarily result from the application of hydrostatic pressure. Upon further compression of GUF-1-OCH_3_ from 0.71 GPa to 4.98 GPa, *ψ* gradually decreases by 3.75(4)° (−4.0%, Fig. 2b, Table S3†). No such behaviour is observed in the native framework, GUF-1-(HCl), up to 0.47 GPa due to the initial intrusion of CH_3_OH into the channels, which limits the framework flexibility as we have observed in Zr MOF systems.^53^

This cluster anion substitution reaction, in effect pressure-induced chemisorption of methanol, is highly unusual in MOFs, but the experimental set-up using the DAC only allows probing of an individual crystal. As we have previously used solid-state NMR spectroscopy to successfully monitor exchange of μ_2_-OH ligands with isotopically enriched water in the related MOF MIL-53(Sc),^38^ we again turned to this technique to determine if the bulk reactivity mirrored that observed in the single crystal. Large-scale samples of the MOF were prepared by acetic acid modulated synthesis to yield GUF-1-(AcOH) and the as-synthesised materials exchanged with either fresh DMF as a control sample, or different isotopologues of methanol: natural abundance CH_3_OH, CD_3_OD (99% ^2^H), and ^13^CH_3_OH (99% ^13^C). A large volume press was used to apply pressure to the samples (see ESI, Section S4†). Each suspension was individually transferred to a sample chamber comprising a 60 mm length of Teflon tubing (ID 8 mm, OD 10 mm) sealed with Teflon caps and Teflon tape. The sample capsule was inserted into a large volume press assembly and a load of 7 tonnes was applied (equivalent pressure = 0.8 GPa, the highest pressure accessible with the equipment).^78^ The samples were held at elevated pressure for a period of 16 h at room temperature (*ca.* 20 °C). For all tested samples, the load on the sample had decreased to ∼6–6.5 tonnes (pressure = 0.69–0.75 GPa) indicating a decrease in sample pressure over the 16 h period. This is frequently observed in other systems and hence is due to a mechanical effect rather than changes to the sample. After this time, the sample was returned to atmospheric pressure over a period of 10 minutes and recovered as a suspension. Control experiments were carried out on identical samples that were exchanged in methanol at ambient pressure for the same time period. The samples are named GUF-1-(solv)-*X*, where solv = DMF or the methanol isotopologue used, and *X* = *am* (ambient) or *P* (pressurised to 0.8 GPa) to denote the pressure used for postsynthetic exchange (see ESI, Table S1†).

Solid-state NMR spectroscopy was then employed to further investigate the nature of the cluster anion substitution process using ^13^C, ^1^H and ^2^H magic angle spinning (MAS) NMR experiments (see ESI, Section S5†). Assignments of the signals from the linker carbon environments are based on ^13^C NMR experiments performed on GUF-1-(DMF)-*am* (see ESI, Section S5.1,† which includes the atom labelling scheme). No resonances corresponding to acetic acid, or acetate acting as a cluster-capping defect, were observed before or after exchange with DMF. Initial ^13^C MAS and cross polarisation (CP) MAS NMR spectra (see ESI, Section S5.2†) acquired for a sample exchanged with unenriched methanol at ambient pressure – GUF-1-(CH_3_OH)-*am* – show five resonances corresponding to the MOF linker at *δ* = 170 (C1, carboxyl carbon), 134 (C2), 132 (C3 and C4), 127 (C5), and 96 ppm (C6, alkyne carbon), as well as three resonances arising from the presence of DMF (*δ* = 161, 35 and 30 ppm). Alongside these peaks is a small additional resonance at 56 ppm, a typical *δ*_iso_ value for the ^13^C nucleus of a OCH_3_ group. This peak remains following calcination of the MOF at 140 °C, 10^−4^ torr for 48 hours, indicating it arises from framework bound OCH_3_ rather than free CH_3_OH within the MOF pores. However, the intensity of this signal is low, and accurate information on the relative percentage of unenriched CH_3_OH exchange taking place within the framework cannot be determined easily from these ^13^C NMR spectra.

**Fig. 4:**
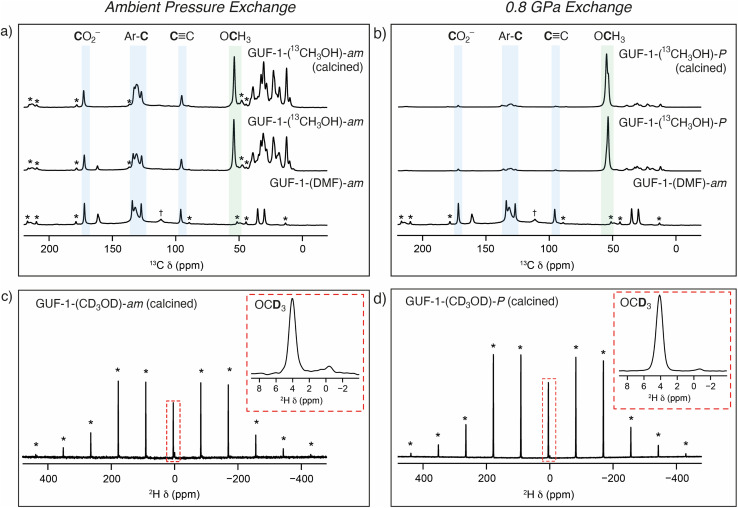
Top: comparative ^13^C (14.1 T, 12.5 kHz) MAS NMR spectra of GUF-1-(DMF)-*am* before and after soaking in ^13^CH_3_OH and calcination at (a) ambient pressure (GUF-1-(^13^CH_3_OH)-*am*), and (b) at 0.8 GPa in the large volume press (GUF-1-(^13^CH_3_OH)-*P*). Resonances shaded in blue correspond to carbon atoms of the EDB^2−^ linker, and the resonances shaded green to the OCH_3_ group coordinated to the SBU (56 ppm). Resonances for ^13^C nuclei of DMF are visible in the spectrum of GUF-1-(DMF)-*am* at 30, 35, and 161 ppm. Resonances between 10 and 50 ppm correspond to a minor impurity introduced by the ^13^CH_3_OH (see ESI†). Bottom: ^2^H (14.1 T, 8 kHz) MAS NMR spectra of (c) GUF-1-(CD_3_OD)-*am* and (d) GUF-1-(CD_3_OD)-*P*, with the resonances assigned to the OCD_3_ group at 4 ppm shown in inserts. Dagger (†) denotes a signal arising from a PTFE 4 mm MAS NMR rotor insert. Asterisks (*) denote spinning sidebands.

Repeating the methanol exchange process with ^13^CH_3_OH (99% ^13^C) enables ^13^C NMR spectra to be acquired with an improved signal-to-noise ratio. This process was performed on two samples of GUF-1-(AcOH), one exchanged under ambient pressure conditions – GUF-1-(^13^CH_3_OH)-*am* – and a second which had been pressurised to 0.8 GPa in the large volume press, GUF-1-(^13^CH_3_OH)-*P*. Both materials were stored in their ^13^CH_3_OH solvent for 6 further days after pressurisation, prior to filtering and packing in MAS NMR rotors. ^13^C and ^1^H MAS NMR spectra were acquired on the freshly filtered materials, and subsequently after calcination at 140 °C, 10^−4^ torr for 48 h. The ^13^C MAS NMR spectra for the materials before and after calcination are shown in Fig. 4a and b, while ^13^C CP MAS and ^1^H MAS NMR spectra are provided in the ESI, Section S5.3.† In order to acquire a quantitative ^13^C MAS NMR spectrum, *T*_1_ relaxation measurements were carried out on GUF-1-(^13^CH_3_OH)-*am* using a saturation recovery experiment, which indicated the alkyne carbon had the slowest relaxation (with *T*_1_ values of 10 and 12 s for the post-soaking and calcined forms, respectively) and thus a recycle interval of 2 minutes was utilised for every ^13^C MAS NMR spectrum. The ^13^C MAS NMR spectrum of GUF-1-(^13^CH_3_OH)-*am* (Fig. 4a) shows significant enhancement of the resonance at 56 ppm, providing further evidence that this peak relates to the exchanged μ_2_-OCH_3_. Fitting and integrating the peaks in the solid-state NMR spectrum, including spinning sidebands, indicates 2.2(2)% of the hydroxyl groups in the framework have been replaced with μ_2_-OCH_3_ following the ambient pressure exchange step. After calcination this value decreases to 1.6(3)%, suggesting a very small portion of free ^13^CH_3_OH was still present in the original material. Additional resonances arise in the ^13^C MAS NMR spectrum between 10 and 50 ppm when the material is exchanged with ^13^CH_3_OH. It is believed that these peaks correspond to a minor impurity in the material, present at 2–3% (w/w), but are only observable when ^13^CH_3_OH is used and would be too weak to observe crystallographically. A more detailed discussion of the possible nature of these signals is included in the ESI, Section S5.3.†

For the sample prepared under high pressure, GUF-1-(^13^CH_3_OH)-*P*, the percentage of μ_2_-OCH_3_ exchange increases significantly to 20(4)%, decreasing slightly to 17(2)% following calcination (Fig. 4b), confirming the significant effect of pressure in enhancing cluster anion exchange by an order of magnitude, and the stability of the μ_2_-OCH_3_ substituent once the pressure is returned to ambient and the CH_3_OH is removed. These values correspond reasonably well with the 33(3)% μ_2_-OCH_3_ exchange observed using crystallography at 0.71 GPa. Powder X-ray diffraction analysis of the samples recovered following solid-state NMR spectroscopy shows that the MOFs remain intact after the bulk scale pressurisation, with some minor differences in relative peak intensities apparent after calcination (see ESI, Section S6†).

To provide additional evidence of μ_2_-OCH_3_ binding to the GUF-1 framework, ^2^H MAS NMR spectra of materials exchanged using CD_3_OD (99% ^2^H) were acquired to investigate any limitations on free rotation of methanol through measurement of the ^2^H quadrupolar coupling constant, *C*_Q_. In general, for ^2^H in a molecule which can rotate isotropically, as in solution, a *C*_Q_ value of 0 would be observed (*i.e.*, the anisotropic quadrupolar interaction would be completely removed). Larger values of *C*_Q_ indicate restricted motion of the molecule, for example if μ_2_-OCH_3_ was bound to the MOF. The ^2^H MAS NMR spectra of the calcined frameworks in Fig. 4c and d, GUF-1-(CD_3_OD)-*am* and GUF-1-(CD_3_OD)-*P*, respectively, show two resonances at 3.9 and −0.5 ppm (insets), corresponding to deuterated μ_2_-OCD_3_ and the previously mentioned minor impurity (see ESI, Section 5.3†), respectively. Fitting of the resonance at 3.9 ppm gives a *C*_Q_ value of 46(3) kHz for the framework exchanged under ambient conditions and 48(3) kHz when exchange is carried out under higher pressure. Both fittings provide an *η*_Q_ value (where *η*_Q_ is a measure of the asymmetry of the quadrupolar interaction)^79^ of 0.0(2) confirming an axially symmetric averaged electric field gradient tensor, as expected for a *C*_3_ rotation. This provides further evidence that the resonance at 3.9 ppm corresponds to μ_2_-OCH_3_ that is bound to the MOF at the SBU rather than present as free CH_3_OH within the framework pores.

## Conclusions

In summary, we have demonstrated that the bridging μ_2_-OH ligands in the MIL-53 topology Sc^iii^ MOF GUF-1 can be exchanged for μ_2_-OCH_3_ ligands through pressure-induced reaction with pore-bound methanol in a process we have termed cluster anion substitution. Up to 98(4)% of the μ_2_-OH groups were seen to exchange crystallographically at 4.98 GPa in a single crystal pressurised in a diamond anvil cell, while solid-state NMR spectroscopy showed 17(2)% exchange in bulk samples subjected to 0.8 GPa in a large volume press, an order of magnitude greater than the 1.6(3)% exchange observed for samples soaked in methanol under ambient conditions. We hypothesise that pressure-induced intrusion of CH_3_OH into the pores of GUF-1 brings the adsorbate into close contact with the framework and results in an enhancement of reactivity due to close confinement of the reagents. These findings provide further evidence of the lability of the bridging hydroxo ligands commonly found in MOF SBUs, beyond the simple μ_2_-OH/μ_2_-^17^OH exchange reported previously in MIL-53(Sc) under hydrothermal conditions, and suggest that CH_3_OH activation of MOFs should be carefully monitored for unintentional reactivity and chemisorption of the solvent. Indeed, refluxing a sample of GUF-1-(DMF)-*am* in CH_3_OH for 16 h results in 46(2)% exchange (see ESI, Section S6.3†); whilst this is lower than the near quantitative exchange achieved at 4.98 GPa, it further demonstrates the lability of the SBU. The presumably associative ligand exchange mechanism could be a proxy not only for solvent-induced structural breakdown of MOFs – for example certain Zr MOFs containing μ_2_-OH ligands at their SBUs are known to be sensitive to CH_3_OH^80–82^ – but also a potential mechanism for small molecule activation, as similar bridging methoxide units at the SBUs of related MOFs have been implicated in catalytic mechanisms.^83^ In addition, formation of terminal Zr–OCH_3_ units at defect sites in Zr MOFs is implicated in their CH_3_OH activation for further functionalisation,^84^ while the presence of μ_2_-OCH_3_ in the SBUs of GUF-1 could explain changes in physical properties observed when related MIL-53 analogues are directly synthesised in CH_3_OH.^85,86^ Our bulk scale pressure measurements identified a low level (∼2%) pore-located impurity in the samples, which we have not currently identified, but whose isotopic enrichment confirms its origin in the ^13^CH_3_OH used to induce reactivity. Future work will seek to identify this material and exploit transient alkoxide coordination for catalytic reactions.

## Data availability

CCDC 2223543–2223556 contain the supplementary crystallographic data. These data can be obtained free of charge from The Cambridge Crystallographic Data Centre; see https://www.ccdc.cam.ac.uk/. The data that support the findings of this study are openly available in University of Glasgow, Enlighten repository at https://doi.org/10.5525/gla.researchdata.1453.†

## Author contributions

R. S. F. and S. A. M. conceived the project. A. J. R. T. and W. L. W. L. synthesised all materials, and carried out all postsynthetic modifications and lab scale characterisation. A. J. R. T., G. F. T., I. P., C. L. H., D. R. A. and M. R. W. (Warren) carried out high-pressure single crystal X-ray diffraction measurements at Diamond Light Source, and G. F. T. and S. A. M. analysed the data. M. R. W. (Ward) and I. D. H. O. carried out bulk scale pressurisation experiments in the high-volume press. Z. H. D. carried out solid-state NMR spectroscopic experiments and analysed the data with R. E. M. and S. E. A. All authors contributed to the preparation of the manuscript, which was initially drafted by A. J. R. T., R. S. F., G. F. T., S. A. M., Z. H. F., and S. E. A.

## Conflicts of interest

The authors declare no competing interests.

## Supplementary Material

SC-014-D3SC00904A-s001

SC-014-D3SC00904A-s002
