# Supplementary material for: Pressure-induced postsynthetic cluster anion substitution in a MIL-53 topology scandium metal–organic framework
Source: Chem Sci. 2023 Jun 19;14(28):7716–24. doi: 10.1039/d3sc00904a (PMC10355111; doi:10.1039/d3sc00904a)
Supplement: SC-014-D3SC00904A-s001 [file SC-014-D3SC00904A-s001.pdf]

Electronic Supplementary Material (ESI) for Chemical Science.
This journal is © The Royal Society of Chemistry 2023

Pressure-Induced Postsynthetic Cluster Anion Substitution in a MIL-53 Topology Scandium Metal-Organic Framework

Alexander J. R. Thom,[a] Gemma F. Turner,[b] Zachary H. Davis,[c] Martin R. Ward,[d] Ignas Pakamorė,[a] Claire L. Hobday,[e] David R. Allan,[f] Mark R. Warren,[f] Wai L. W. Leung,[a] Iain D. H. Oswald,[d] Russell E. Morris,[c] Stephen A. Moggach,[b*] Sharon E. Ashbrook[c*] and Ross S. Forgan[a*]

[a]WestCHEM School of Chemistry, University of Glasgow, Joseph Black Building, University Avenue, Glasgow G12 8QQ, UK.
Email: ross.forgan@glasgow.ac.uk

[b]School of Molecular Sciences, The University of Western Australia, 35 Stirling Highway, Crawley, Perth, Western Australia, 6009, Australia.
Email: stephen.moggach@uwa.edu.au

[c] EaStCHEM School of Chemistry and Centre of Magnetic Resonance, University of St Andrews, St Andrews KY16 9ST, UK.
Email: sema@st-andrews.ac.uk

[d]Strathclyde Institute of Pharmacy & Biomedical Sciences (SIPBS), University of Strathclyde, 161 Cathedral Street, Glasgow, G4 0RE, UK.

[e]EaStCHEM School of Chemistry and Centre for Science at Extreme Conditions, The University of Edinburgh, King's Buildings, David Brewster Road, Edinburgh EH9 3FJ, UK.

[f]Diamond Light Source Ltd, Harwell Science and Innovation Campus, Didcot, Oxfordshire OX11 0DE, UK.

SUPPORTING INFORMATION

Contents

S1. General Experimental Remarks S3

S2. Synthesis S5

S2.1. Ligand Synthesis S5

S2.2. GUF-1-(HCl) Synthesis S5

S2.3. Synthesis of GUF-1-(AcOH) S5

S3. Single Crystal X-Ray Diffraction S7

S3.1. Ambient Pressure S7

S3.2. High Pressure S7

S4. Bulk Pressurisation of GUF-1-(AcOH) S14

S5. Solid-State NMR Spectroscopy S15

S5.1 GUF-1-(DMF)-*am* S15

S5.2 GUF-1-(CH_3OH)-*am* S17

S5.3 GUF-1-($^{13}CH_3OH$)-*am* and GUF-1-($^{13}CH_3OH$)-*P* – Minor Impurity S18

S6. Scale-Up and Bulk Characterisation S24

S6.1. GUF-1-(AcOH) Control S24

S6.2. Analysis of Samples used in Solid-State NMR Measurements S31

S6.3. GUF-1-(CH_3OH)-*reflux* S33

S7. References S35

S1. General Experimental Remarks

Chemicals and solvents were purchased from Alfa Aesar, Fluorochem, Tokyo Chemical Industry, Sigma-Aldrich, Strem and VWR and used without further purification.

Single Crystal X-ray diffraction (SCXRD): Ambient pressure single crystal diffraction data were collected using a Bruker APEX II diffractometer, with graphite-monochromated, MoKα (λ = 0.71073 nm) radiation. High pressure single crystal diffraction data were collected at the I19-2 chemical crystallography beamline at Diamond Light Source (Section S3).

Powder X-ray diffraction (PXRD): PXRD measurements were carried out at the University of Glasgow at 298 K using a Rigaku Miniflex diffractometer (λ (CuKα(mean)) = 1.54183 Å) on a zero-background sample plate on a rotating sample stage. Data were collected from 3–45° at a step size of 0.01°.

Gas Uptake: N_2 adsorption and desorption isotherms up to 1 bar were carried out at 77 K on a Quantachrome Autosorb iQ gas sorption analyser. Samples were degassed under vacuum at 150 °C for 20 h using the internal turbo pump. BET surface areas were calculated from the isotherms using the Micropore BET Assistant in the Quantachrome ASiQwin operating software.

Large Volume Press (LVP): Bulk solvent exchange of GUF-1-(AcOH) samples was performed using a large volume press apparatus.[S1] The press comprises a copper beryllium pressure cell that accommodates a Teflon sample chamber (10 mm OD, 8 mm ID). Samples of GUF-1-(AcOH) were suspended in the relevant solvent system (CH_3OH, CD_3OD, or $^{13}CH_3OH$) and transferred to the cylindrical Teflon sample chamber and sealed using Teflon tape and Teflon caps. The pressure cell was sealed before applying a 7-ton load to the sample chamber (equivalent to 0.8 GPa) by means of a hydraulic press. The samples were held at elevated pressure overnight (16 h, at room temperature), where the load on the sample had reduced to 6–6.5 tonnes (0.69–0.75 GPa) before recovering the sample back to ambient conditions. The final pressure is the value we take as the pressure on the system. The recovered sample was retained as a suspension in a sealed vial for subsequent analysis.

Nuclear Magnetic Resonance (NMR) Spectroscopy: Liquid-state NMR spectra were recorded on either a Bruker Avance III 400 MHz spectrometer or a Bruker Avance I 500 MHz spectrometer and referenced to residual solvent peaks. MOF samples were digested with D_2SO_4 in DMSO-d_6. Solid-state NMR spectra were acquired using a Bruker Avance III 600 MHz spectrometer equipped with a 14.1 T wide-bore magnet. Samples were packed into a 4

mm ZrO_2 rotor and sealed with a ZrO_2 cap. Magic angle spinning (MAS) NMR spectra were acquired at spinning speeds of 12.5 kHz (^{1}H and ^{13}C) and 8 kHz (^{2}H) using a conventional 4 mm HX probe. Spectra were acquired at Larmor frequencies of 600.13 MHz, 150.87 MHz and 92.12 MHz for ^{1}H, ^{13}C and ^{2}H, respectively. Radiofrequency field strengths were 100 kHz (^{1}H), 91 kHz (^{13}C) and 62.5 kHz (^{2}H). Spectra are referenced to $Si(CH_3)_4$ for ^{1}H and ^{13}C (using a secondary reference of L-alanine ($\delta(N\mathbf{H}_3)$ = 8.5 ppm and $\delta(\mathbf{C}H_3)$ = 20.5 ppm)) and ^{2}H (using a secondary reference of D-oxalic acid ($\delta(O\mathbf{D})$ = 16.5 ppm)). ^{1}H MAS NMR spectra were acquired using a rotor-synchronised spin-echo pulse sequence. ^{13}C MAS NMR spectra were acquired either using direct polarisation or cross polarisation (CP)[S2] from ^{1}H using a 2.5 ms contact pulse (ramped for ^{1}H). TPPM-15[S3] ^{1}H decoupling (100 kHz) was applied during acquisition of all ^{13}C MAS NMR spectra. ^{1}H/^{13}C heteronuclear correlation (HETCOR) spectra were recorded using CP from ^{1}H as described above and are the result of averaging 144 transients for each of 138 t_1 increments of 80 μs. Two-dimensional refocused INADEQUATE spectra were acquired using the pulse sequence in reference S4, with a rotor-synchronised evolution period of 1.84 ms, and are the result of averaging 96 transients for each of 230 t_1 increments of 40 μs.

S2. Synthesis

S2.1. Ligand Synthesis

The ligand 4,4'-(ethyne-1,2-diyl)dibenzoic acid (EDB-H_2) was prepared according to our previously reported method.[S5]

S2.2. GUF-1-(HCl) Synthesis

Scandium nitrate tetrahydrate (30 mg, 0.1 mmol) and EDB-H_2 (26.4 mg, 0.1 mmol) were added to a 25 ml Pyrex vial with *N*,*N*-dimethylformamide (DMF 2.3 ml, 30 mmol). HCl (61 μL, 2 mmol) was added, the mixture sonicated and placed in an oven at 100 ºC for 24 h. After 24 h, the vial was removed from the oven and allowed to cool to room temperature, yielding light pink crystals suitable for single crystal X-ray diffraction, named GUF-1-(HCl).

S2.3. Synthesis of GUF-1-(AcOH)

Scandium nitrate tetrahydrate (90 mg, 0.3 mmol) and EDB-H_2 (79.2 mg, 0.3 mmol) were added to a 50 ml Pyrex vial with DMF (6.9 ml, 90 mmol). Acetic acid (AcOH, 171 μL, 6 mmol) was added, the mixture sonicated and placed in an oven at 100 ºC for 24 h. After 24 h, the vial was removed from the oven and allowed to cool to room temperature. The contents of three individual vials (typically 50-100 mg of MOF) were combined and transferred to a 50 ml centrifuge tube and washed once with either fresh DMF, natural abundance CH_3OH, CD_3OD (99% ^2H) or $^{13}CH_3OH$ (99% ^{13}C) once, depending on the experiment, then replenished with fresh solvent and left to stand. The naming scheme for the samples is given in Table S1. The samples are named GUF-1-(*solv*)-*X*, where *solv* = the solvent used for exchange, and *X* = *am* (ambient), *P* (pressurised to 0.8 GPa for 16 h, see Section S4) or *reflux* (refluxed in a round bottom flask for 16 h) to denote the conditions used for postsynthetic exchange. Solid-state NMR spectra were collected for materials in both their post-exchanged state and after subsequent calcination. Materials were calcined by heating to 140 °C, under a pressure of 10^{-4} Torr for 48 h. The calcined samples were sealed under an argon atmosphere before packing into a MAS NMR rotor for analysis.

Table S1. Sample naming scheme for bulk solvent-exchanged samples of GUF-1-(AcOH).

**Name**	**Pore Solvent**	**Exchange Conditions**
GUF-1-(DMF)-*am*	DMF (natural abundance)	Ambient ("as-synthesised")
GUF-1-(CH_3OH)-*am*	CH_3OH (natural abundance)	Ambient
GUF-1-(CH_3OH)-*P*	CH_3OH (natural abundance)	0.8 GPa (16 h)
GUF-1-(CH_3OH)-*reflux*	CH_3OH (natural abundance)	Reflux (16 h)
GUF-1-(CD_3OD)-*am*	CD_3OD (99% ^2H)	Ambient
GUF-1-(CD_3OD)-*P*	CD_3OD (99% ^2H)	0.8 GPa (16 h)
GUF-1-($^{13}CH_3OH$)-*am*	$^{13}CH_3OH$ (99% ^{13}C)	Ambient
GUF-1-($^{13}CH_3OH$)-*P*	$^{13}CH_3OH$ (99% ^{13}C)	0.8 GPa (16 h)

S3. Single Crystal X-Ray Diffraction

S3.1. Ambient Pressure

A single crystal of GUF-1-(HCl) was characterised by single crystal X-ray diffraction under ambient conditions of pressure and temperature using a Bruker APEX II diffractometer, with graphite-monochromated, Mo Kα (λ = 0.71073 nm) radiation. Diffraction data were integrated and reduced using SAINT V8.40B.[S6]

The crystal structure was solved using ShelXT[S7] and refined using ShelXL[S8] in Olex2.[S9] Thermal similarity restraints were applied to the EDB^{2-} ligand, and phenyl rings were restrained to planarity. The hydrogen atom on the hydroxide bridge was placed by a Fourier difference map, with the O–H distance restrained to 0.875 Å. All other hydrogen atoms were placed geometrically and constrained to ride on their host atoms.

Crystal data for GUF-1-(HCl) Room temperature ambient pressure structure: $Sc_2C_{32}H_{18}O_{10}\cdot_{1.2}(C_3H_7NO)$, M_r = 713.67, crystal dimensions 0.15 x 0.04 x 0.04 mm, Orthorhombic a = 7.3054 (5), b = 26.5207 (17), c = 11.7550 (9) Å, V = 2277.5 (3) Å^3, T = 273 K, space group *Cmme*, Z = 2, 31343 measured reflections, 1562 unique (R_{int} = 0.056), which were used in refinement calculations. The final R_1 = 0.090 for 1486 observed data [$F^2 > \sigma(F^2)$] and $wR_2(F^2)$ = 0.268 (all data).

S3.2. High Pressure

Single crystals of GUF-1-(HCl) were loaded individually into a miniature Merrill-Bassett diamond anvil cell,[S10] equipped with 600 µm culet Boehlar-Almax diamond anvils, a half-opening angle of 38°, tungsten carbide backing seats and a pre-indented tungsten gasket. Crystals were transferred in their DMF solvents, to maintain crystallinity, under a regular atmosphere. The sample chamber was then filled with a pressure-transmitting medium of either a perfluorinated oil, Fluorinert® FC-70, or HPLC grade CH_3OH. The former was used to assess direct compression of the framework, while the latter was used to assess possible pressure-induced adsorption of CH_3OH into the framework pores. CH_3OH is not expected to freeze at the pressures used, and the absence of any diffraction peaks across our measurements confirms that no crystallisation takes place and so isostatic pressurisation is maintained. A ruby sphere was used to calibrate the pressure in the sample chamber using the ruby fluorescence method.[S12]

High-pressure single crystal diffraction data were collected using synchrotron radiation (λ = 0.4589 nm) on beamline I19-2 at Diamond Light Source, Rutherford Appleton Laboratory, up to a maximum of 4.98 GPa in steps of ~0.5 GPa. A data collection strategy based on Dawson *et al.*[S13] was used, using a step size of 0.2° and an exposure time of 0.2 s. Diffraction data were integrated in CrysAlis Pro,[S14] and corrected for absorption effects using SADABS.[S15]

Crystal structures were solved using ShelXT[S7] and refined using ShelXL[S8] in Olex2.[S9] The structure model determined at ambient pressure was used as the starting coordinates for the first high-pressure structure. For all subsequent high-pressure data, the coordinates from the previous high-pressure structural model data were imported and used as the starting coordinates. The geometry of the EDB^{2-} ligand was restrained to that of the ambient structure, with benzyl groups being restrained to a hexagonal geometry and planarity. All other bond lengths and 1,3-distances in the ligand were restrained to those measured under ambient pressure conditions. All metal-ligand bonds were refined freely. Thermal similarity restraints were applied to the ligands. Guest molecules were refined with isotropic displacement parameters.

For the methoxide-exchanged structure, GUF-1-(OCH_3), the carbon-atom of the methoxide bridge was initially refined with free occupancy, before being fixed to a stable value. This was necessary as the low completeness of the diffraction data (70% to 80%) meant that the partially-occupied methoxide was prone to instability. The hydrogen atom on the hydroxide bridge was placed by a Fourier difference map, with the O–H distance restrained to 0.875 Å. All other hydrogen atoms were placed geometrically and constrained to ride on their host atoms.

Separately, the guest content was also treated as diffuse electron density using the SQUEEZE[S16] algorithm in PLATON[S17] with the ordered guest excluded from the structure model. H-atoms were placed geometrically and constrained to ride on their host-atoms. Structural data were extracted using PLATON[S17] and Mercury software.[S18]

Pertinent crystallographic data are provided in Tables S2 and S3.

CCDC 2223543–2223556 contain the supplementary crystallographic data. These data can be obtained free of charge from The Cambridge Crystallographic Data Centre; see https://www.ccdc.cam.ac.uk/.

Table S2. Unit cell volume, and refined occupancy the methoxide bridge, μ_2-OCH_3, and pore volumes in GUF-1-(HCl) and GUF-1-OCH_3 during hydrostatic compression in a pressure-transmitting medium of MeOH.

***P* / GPa**	**Pore 1 *V* / Å^3 [b]**	**Pore 2 *V* / Å^3 [c]**	**Electrons [d]**	**ψ (°) [e]**	**w/h[f]**
0.00[a]	270	270	56	82.40 (3)	1.09
0.23	273	296	68	96.60 (3)	1.03
0.47	274	297	64	97.41 (3)	1.04
0.71	274	258	63	94.30 (2)	0.85
1.61	261	254	65	95.18 (2)	0.82
2.13	252	247	73	93.28 (2)	0.81
2.61	240	246	81	92.19 (2)	0.80
2.84	248	247	78	91.82 (2)	0.79
3.20	243	242	74	91.80 (2)	0.79
3.45	235	238	83	92.08 (2)	0.78
3.85	230	235	84	91.74 (2)	0.77
4.11	222	236	76	92.12 (2)	0.77
4.60	222	230	57	90.27 (2)	0.76
4.98	216	229	77	90.55 (3)	0.75

[a]Separate crystal, measured at ambient pressure at 273 K.

[b]Volume of 'vacant' channel from PLATON SQUEEZE[S16] with the ordered guest excluded from the structure model.

[c]Volume of μ_2-OH/OMe decorated channel from PLATON SQUEEZE[S16] with the ordered guest excluded from the structure model.

[d]Number of electrons in the μ_2-OH/ μ_2-OCH_3 decorated channel.

[e]"Hinge" angle of the wine-rack structure, defined as the angle between the planes of neighbouring EDB^{2-} ligands

[f]Width to height ratio of the μ_2-OH/μ_2-OCH_3 decorated channel.

Table S3. Crystallographic data for a colourless, block-shaped single crystal of GUF-1-(HCl) and GUF-1-OCH_3 during hydrostatic compression in a pressure-transmitting medium of methanol. All structures are collected at *T* = 298 K on beamline I19-2 at Diamond Light Source (United Kingdom). Continued overleaf…

	**Ambient**	**0.23 GPa**	**0.47 GPa**	**0.71 GPa**
Material	GUF-1-(HCl)	GUF-1-(HCl)	GUF-1-(HCl)	GUF-1-OCH_3
Chemical formula	$Sc_2C_{32}H_{18}O_{10}$·1.2(C_3H_7NO)	$Sc_2C_{32}H_{18}O_{10}$·1.6(C_3H_7NO)·0.2 (CH_4O)	$Sc_2C_{32}H_{18}O_{10}$·(C_3H_7NO)·1.3(CH_4O)	$Sc_2C_{32.5}H_{19.5}O_{10}$·2.8($CH_4O$)·0.9($H_2O$)
M_r	740.1	756.12	768.3	723.19
T / K	273	298	298	298
Crystal system, space group	Orthorhombic, *Cmme*	Orthorhombic, *Cmme*	Orthorhombic, *Cmme*	Orthorhombic, *Cmme*
a, b, c / Å	7.3054(5), 26.5207(17), 11.7550(9)	7.3533 (15), 26.584 (5), 11.879 (2)	7.3205 (15), 26.609 (5), 11.922 (2)	7.3445 (19), 23.984 (3), 13.1770 (13)
V / Å^3	2277.5(3)	2322.2 (8)	2322.2 (8)	2321.1 (7)
Z	2	2	2	2
ρ / g cm^{-3}	1.04	1.081	1.099	1.035
Radiation type	Mo *Kα*	Synchroton, λ = 0.4589 Å	Synchrotron, λ = 0.4589 Å	Synchrotron, λ = 0.4589 Å
μ / mm^{-1}	0.35	0.34	0.34	0.34
Crystal size / mm	0.15 × 0.04 × 0.04	0.15 × 0.04 × 0.04	0.15 × 0.04 × 0.04	0.15 × 0.04 × 0.04
Absorption correction	Multi-scan SADABS	Multi-scan SADABS	Multi-scan SADABS	Multi-scan SADABS
T_{min}, T_{max}	0.676, 0.746	0.547, 0.744	0.366, 0.744	0.312, 0.425
No. of measured, independent and observed reflections	31343, 1562, 1486	1408, 478, 320	1002, 370, 229	482, 482, 395
R_{int}	0.056	0.075	0.116	0.133
θ_{max} (°)	28.3	16.7	13.5	13.4
$(\sin \theta/\lambda)_{max}$ / Å^{-1}	0.668	0.625	0.510	0.476
$R[F^2 > 2\sigma(F^2)]$, $wR(F^2)$, S	0.061, 0.2179, 1.15	0.108, 0.305, 1.18	0.137, 0.370, 1.30	0.136, 0.439, 2.05
No. of reflections	1562	478	370	482
No. of parameters	71	56	56	58
No. of restraints	8	34	34	36
H-atom treatment	H atoms treated by a mixture of independent and constrained refinement	H atoms treated by a mixture of independent and constrained refinement	H atoms treated by a mixture of independent and constrained refinement	H atoms treated by a mixture of independent and constrained refinement
$\Delta\rho_{max}$, $\Delta\rho_{min}$ / e Å^{-3}	1.88, −0.46	0.38, −0.39	0.46, −0.48	1.06, −0.49

Table S3. Crystallographic data for a colourless, block-shaped single crystal of GUF-1-(OCH_3) during hydrostatic compression in a pressure-transmitting medium of methanol. All structures are collected at T = 298 K on beamline I19-2 at Diamond Light Source (United Kingdom). Continued overleaf…

	**1.61 GPa**	**2.13 GPa**	**2.61 GPa**	**2.84 GPa**
Material	GUF-1-OCH_3	GUF-1-OCH_3	GUF-1-OCH_3	GUF-1-OCH_3
Chemical formula	$Sc_2C_{33.9}H_{22.2}O_{10}$·3.5($CH_4O$)·1.9($H_2O$)	$Sc_2C_{33.4}H_{21.1}O_{10}$·4.1($CH_4O$)·1.4($H_2O$)	$Sc_2C_{33.7}H_{21.6}O_{10}$·4.1($CH_4O$)·1.7($H_2O$)	$Sc_2C_{33.4}H_{21.2}O_{10}$·4.1($CH_4O$)·1.4($H_2O$)
M_r	786.66	829.57	837.71	833.27
T / K	298	298	298	298
Crystal system, space group	Orthorhombic, *Cmme*	Orthorhombic, *Cmme*	Orthorhombic, *Cmme*	Orthorhombic, *Cmme*
a, b, c / Å	7.3292 (16), 23.332 (3), 13.3614 (11)	7.290(3), 22.957(4), 13.4286(14)	7.241(4), 22.814(7), 13.473(3)	7.213(2), 22.789(3), 13.4966(13)
V / Å^3	2284.9 (6)	2247.4(9)	2225.6(15)	2218.6(7)
Z	2	2	2	2
ρ / g cm^{-3}	1.143	1.226	1.250	1.247
Radiation type	Synchrotron, λ = 0.4589 Å	Synchrotron, λ = 0.4589 Å	Synchroton, λ = 0.4589 Å	Synchrotron, λ = 0.4589 Å
μ / mm^{-1}	0.35	0.36	0.37	0.37
Crystal size / mm	0.15 × 0.04 × 0.04	0.15 × 0.04 × 0.04	0.15 × 0.04 × 0.04	0.15 × 0.04 × 0.04
Absorption correction	Multi-scan SADABS	Multi-scan SADABS	Multi-scan SADABS	Multi-scan SADABS
T_{min}, T_{max}	0.487, 0.744	0.177, 0.744	0.021, 0.744	0.582, 0.744
No. of measured, independent and observed reflections	6455, 887, 679	3192, 325, 265	3786, 331, 245	4581, 420, 358
R_{int}	0.097	0.191	0.190	0.078
θ_{max} (°)	16.6	11.7	11.7	12.8
(sin θ/λ)$_{max}$ / Å^{-1}	0.622	0.441	0.440	0.481
$R[F^2 > 2\sigma(F^2)]$, $wR(F^2)$, S	0.130, 0.440, 1.89	0.143, 0.430, 1.91	0.114, 0.363, 1.47	0.111, 0.364, 1.66
No. of reflections	887	330	331	418
No. of parameters	34	34	34	34
No. of restraints	28	28	28	28
H-atom treatment	H atoms treated by a mixture of independent and constrained refinement	H atoms treated by a mixture of independent and constrained refinement	H atoms treated by a mixture of independent and constrained refinement	H atoms treated by a mixture of independent and constrained refinement
$\Delta\rho_{max}$, $\Delta\rho_{min}$ / e Å^{-3}	1.36, −0.90	0.54, −0.51	0.46, −0.50	0.65, −0.42

Table S3. Crystallographic data for a colourless, block-shaped single crystal of GUF-1-(OCH_3) during hydrostatic compression in a pressure-transmitting medium of methanol. All structures are collected at *T* = 298 K on beamline I19-2 at Diamond Light Source (United Kingdom). Continued overleaf…

	**3.20 GPa**	**3.45 GPa**	**3.85 GPa**	**4.11 GPa**
Material	GUF-1-OCH_3	GUF-1-OCH_3	GUF-1-OCH_3	GUF-1-OCH_3
Chemical formula	$Sc_2C_{33.7}H_{21.6}O_{10}$·3.9($CH_4O$)·1.7($H_2O$)	$Sc_2C_{33.6}H_{21.2}O_{10}$·3.7($CH_4O$)·1.6($H_2O$)	$Sc_2C_{33.4}H_{20.9}O_{10}$·3.9($CH_4O$)·1.4($H_2O$)	$Sc_2C_{33.8}H_{21.8}O_{10}$·3.7($CH_4O$)·1.8($H_2O$)
M_r	832.03	821.49	821.59	828.82
T / K	298	298	298	298
Crystal system, space group	Orthorhombic, *Cmme*	Orthorhombic, *Cmme*	Orthorhombic, *Cmme*	Orthorhombic, *Cmme*
a, *b*, *c* / Å	7.180(2), 22.587(4), 13.5541(13)	7.1429(17), 22.463(3), 13.5504(12)	7.1069(18), 22.297(4), 13.5858(16)	7.0769(14), 22.232(4), 13.6130(15)
V / Å^3	2198.2(7)	2174.2(7)	2152.8(7)	2141.7(6)
Z	2	2	2	2
ρ / g cm^{-3}	1.257	1.255	1.267	1.285
Radiation type	Synchrotron, λ = 0.4589 Å	Synchrotron, λ = 0.4589 Å	Synchrotron, λ = 0.4589 Å	Synchrotron, λ = 0.4589 Å
μ / mm^{-1}	0.37	0.37	0.38	0.38
Crystal size / mm	0.15 × 0.04 × 0.04	0.15 × 0.04 × 0.04	0.15 × 0.04 × 0.04	0.15 × 0.04 × 0.04
Absorption correction	Multi-scan SADABS	Multi-scan SADABS	Multi-scan SADABS	Multi-scan SADABS
T_{min}, T_{max}	0.485, 0.744	0.680, 0.744	0.539, 0.745	0.533, 0.744
No. of measured, independent and observed reflections	4563, 434, 343	4239, 386, 315	4302, 427, 315	4190, 388, 299
R_{int}	0.102	0.093	0.123	0.111
θ_{max} (°)	12.8	12.2	12.6	12.1
$(\sin \theta/\lambda)_{max}$ / Å^{-1}	0.481	0.460	0.477	0.458
$R[F^2 > 2\sigma(F^2)]$, $wR(F^2)$, S	0.111, 0.364, 1.66	0.116, 0.357, 1.60	0.130, 0.426, 1.81	0.090, 0.270, 1.10
No. of reflections	418	383	428	388
No. of parameters	34	34	32	34
No. of restraints	28	28	28	28
H-atom treatment	H atoms treated by a mixture of independent and constrained refinement	H atoms treated by a mixture of independent and constrained refinement	H atoms treated by a mixture of independent and constrained refinement	H atoms treated by a mixture of independent and constrained refinement
$\Delta\rho_{max}$, $\Delta\rho_{min}$ / e Å^{-3}	0.65, −0.42	0.55, −0.45	0.60, −1.07	0.49, −0.33

Table S3. Crystallographic data for a colourless, block-shaped single crystal of GUF-1-(OCH_3) during hydrostatic compression in a pressure-transmitting medium of methanol. All structures are collected at T = 298 K on beamline I19-2 at Diamond Light Source (United Kingdom).

	**4.60 GPa**	**4.98 GPa**
Material	GUF-1-OCH_3	GUF-1-OCH_3
Chemical formula	$Sc_2C_{33.7}H_{21.8}O_{10}$·2.5($CH_4O$)·1.7($H_2O$)	$Sc_2C_{34}H_{22.2}O_{10}$·3.6(CH_4O)·1.9(H_2O)
M_r	788.87	830.23
T / K	298	298
Crystal system, space group	Orthorhombic, *Cmme*	Orthorhombic, *Cmme*
a, b, c / Å	7.0380(15), 22.061(6), 13.652(2)	7.0143(12), 21.941(7), 13.6972(19)
V (Å^3)	2119.8(8)	2108.0(8)
Z	2	2
ρ / g cm^{-3}	1.236	1.308
Radiation type	Synchrotron, λ = 0.4589 Å	Synchrotron, λ = 0.4589 Å
μ / mm^{-1}	0.38	0.39
Crystal size / mm	0.15 × 0.04 × 0.04	0.15 × 0.04 × 0.04
Absorption correction	Multi-scan SADABS	Multi-scan SADABS
T_{min}, T_{max}	0.429, 0.744	0.390, 0.744
No. of measured, independent and observed reflections	4470, 427, 300	3488, 309, 221
R_{int}	0.127	0.354
θ_{max} (°)	12.8	11.7
$(\sin \theta/\lambda)_{max}$ / Å^{-1}	0.481	0.441
$R[F^2 > 2\sigma(F^2)]$, $wR(F^2)$, S	0.100, 0.293, 1.10	0.111, 0.333, 1.22
No. of reflections	427	309
No. of parameters	34	34
No. of restraints	28	28
H-atom treatment	H atoms treated by a mixture of independent and constrained refinement	H atoms treated by a mixture of independent and constrained refinement
$\Delta\rho_{max}$, $\Delta\rho_{min}$ / e Å^{-3}	0.42, −0.32	0.56, −0.33

S4. Bulk Pressurisation of GUF-1-(AcOH)

To apply the pressure for the enhanced CH_3OH exchange, the material was left in its solvent and each suspension was individually transferred to a sample chamber comprising a 60 mm length of Teflon tubing (ID 8 mm, OD 10 mm) sealed with Teflon caps and Teflon tape. The sample capsule was then inserted in to a large volume press assembly and a load of 7 tonnes was applied (equivalent pressure = 0.8 GPa).[S1] The samples were held at elevated pressure for a period of 16 h at room temperature (*ca.* 20 °C). For all tested samples, the load on the sample had decreased to ~6-6.5 tonnes (pressure = 0.69 – 0.75 GPa) indicating a decrease in sample pressure over the 16 h period. After this time, the sample was returned to atmospheric pressure and the sample recovered as a suspension. Materials were stored in their solvent for 6 further days after pressurisation, prior to filtering and packing in MAS NMR rotors.

S5. Solid-State NMR Spectroscopy

Solid-state NMR spectroscopy was used to probe the substitution process, and quantify OCH_3 exchange through quantitative ^{13}C MAS NMR spectra. Peak assignments in the organic EDB^{2-} linker were made according to the ligand numbering scheme shown in Figure S1.

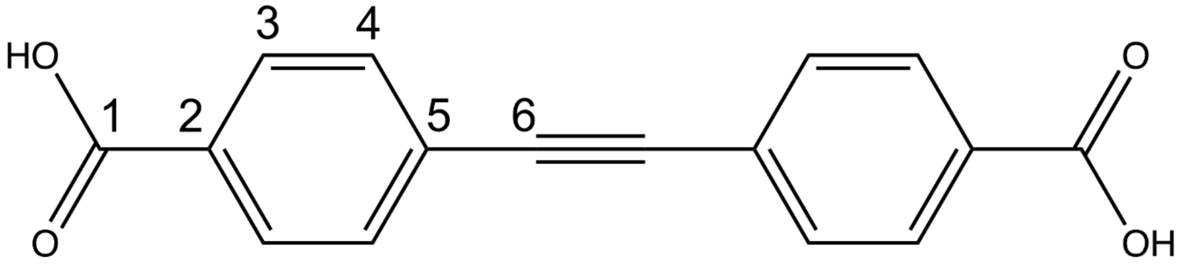

Figure S1. Schematic showing the numbering system used to identify atoms within the organic linker.

S5.1 GUF-1-(DMF)-*am*

The ^{13}C CP MAS NMR spectra acquired with different contact times of GUF-1-(DMF)-*am* framework in its as-made form, containing DMF within the pores, are shown in Figures S2a-2c. Note that spectra acquired using CP are inherently non quantitative as the intensity depends on the spatial proximities of ^{1}H and ^{13}C. C1 and C6 can be assigned simply of the basis on chemical shifts to the resonances at 170 ppm and 96 ppm respectively. Assignment of C2 through C5 was determined by varying the contact time of the ^{13}C CP NMR experiment, in which signal enhancement of the resonance at 132 ppm was observed at a shorter time (0.25 ms), resulting in assignment of this peak to C3 and C4. Following this, C2 and C5 were assigned based on chemical shifts to the resonances at 134 ppm and 127 ppm respectively. The presence of DMF is clearly seen through the three peaks at 161, 35, and 30 ppm. Closer inspection of the resonance at 161 ppm shows, at longer contact times, the overlap of two signals, indicating the presence of two crystallographically unique DMF molecules, which is also suggested by the ^{1}H MAS NMR spectrum, Figure S2d, in which two resonances (6.8 and 6.7 ppm) can be assigned to the aldehyde.

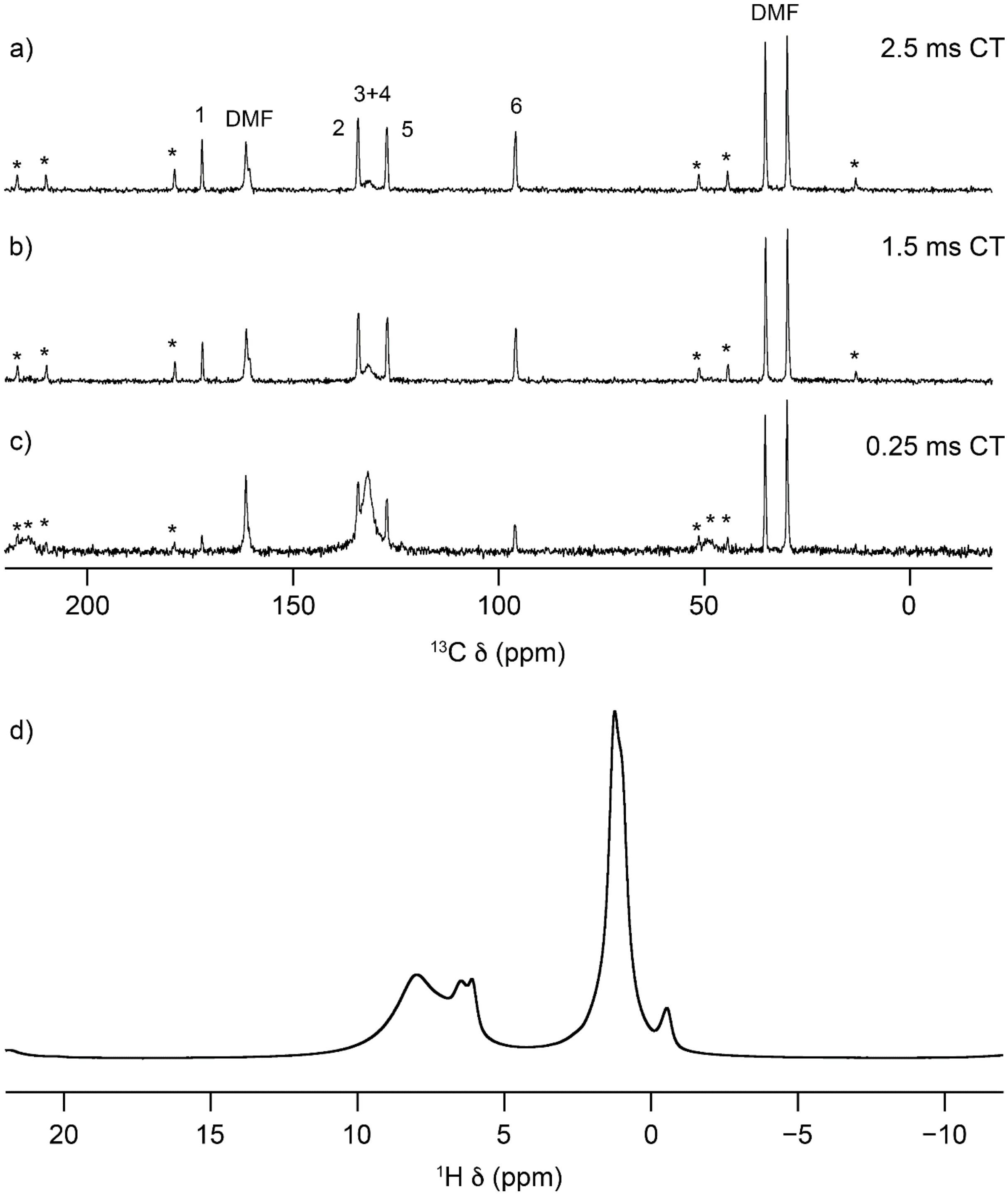

Figure S2. Solid-state NMR spectra of GUF-1-(DMF)-*am*. a), b) and c) show ^{13}C CP (14.1 T, 12.5 kHz) MAS NMR spectra, with various contact times (CT) as labelled on the spectra, showing the assignments of the six unique carbons in the organic linker (numbering scheme in Figure S1). Asterisks (*) denote spinning sidebands. d) ^{1}H (14.1 T, 12.5 kHz) MAS NMR spectra of GUF-1-(DMF)-*am*.

Two overlapping peaks at 1.1 ppm (evidenced as a shoulder on the resonance) are assigned to the methyl groups in DMF. The protons on the benzene ring (~7.9 ppm) cannot be resolved at this relatively slow MAS rate. The bridging hydroxyl groups of the framework are also not resolved. Based on information from related systems, such as in dehydrated MIL-53(Sc) where the hydroxyl resonance appears a ~2 ppm,[S19] it can be determined in GUF-1-(DMF)-*am* that this peak is overlapped with the methyl ^{1}H DMF signals present at 1.1 ppm.. The origin of the low intensity signal resonance at −0.5 ppm is unknown, but is likely to arise from an impurity (see later). ^{1}H MAS NMR assignments are based on ^{1}H−^{13}C HETCOR NMR experiments carried out on GUF-1-($^{13}CH_3OH$)-*am* (see Section S5.3).

S5.2 GUF-1-(CH_3OH)-*am*

Figure S3 shows ^{13}C and ^{1}H MAS NMR spectra of GUF-1-(CH_3OH)-*am*, a sample exchanged with natural abundance CH_3OH under ambient conditions, both post soaking and after calcination. Resonances corresponding to carbon nuclei in the organic linker appear at similar chemical shifts to those seen for the as-made, DMF loaded sample. The ^{13}C MAS NMR spectra show clearly the presence of DMF even after the exchange procedure. An additional resonance is observed at 56 ppm corresponding to the μ_2-OCH_3 group which has substituted some of the μ_2-OH units of GUF-1. The resonance remains following the calcination procedure while the resonances for DMF are lost (this appears much more clearly in the ^{13}C MAS NMR spectrum of GUF-1-($^{13}CH_3OH$)-*am* shown in the next section), indicating that it is indeed a methoxide group bound to the framework rather than free CH_3OH within the MOF pores. The level of exchange is too small to accurately determine the percentage of μ_2-OCH_3 attached to the framework in the quantitative ^{13}C NMR spectrum.

The ^{1}H MAS NMR spectrum shows peaks corresponding to the benzene ring (7.9 ppm), DMF (aldehyde and methyl groups at 6.8 and 1.1 ppm), and an unknown impurity peak (at –0.5 ppm). It should be noted that unlike GUF-1-(DMF)-*am*, there is no splitting of the signals attributed to the aldehyde ^{13}C and ^{1}H, suggesting only one crystallographically distinct DMF molecule is present. There is an additional resonance at 3.6 ppm, resulting from μ_2-OCH_3 bound to the MOF. Removal of all residual DMF occurs during the calcination procedure, as indicated by the absence of these resonances in the NMR spectra of the calcined materials. Of note, the impurity peak observed in the ^{1}H MAS NMR spectrum remains following this procedure, suggesting it is part of a larger molecule which is not easily removed from the framework. Finally, it can be seen that the lineshapes in the ^{13}C MAS NMR spectra broaden

following calcination. This could be attributed to removal of dynamic solvent, which could result in slower relaxation, and thus a broadening of these resonances, or could result from minor variation in the pore structure once the solvent molecules are removed, leading to a range of chemical shifts.

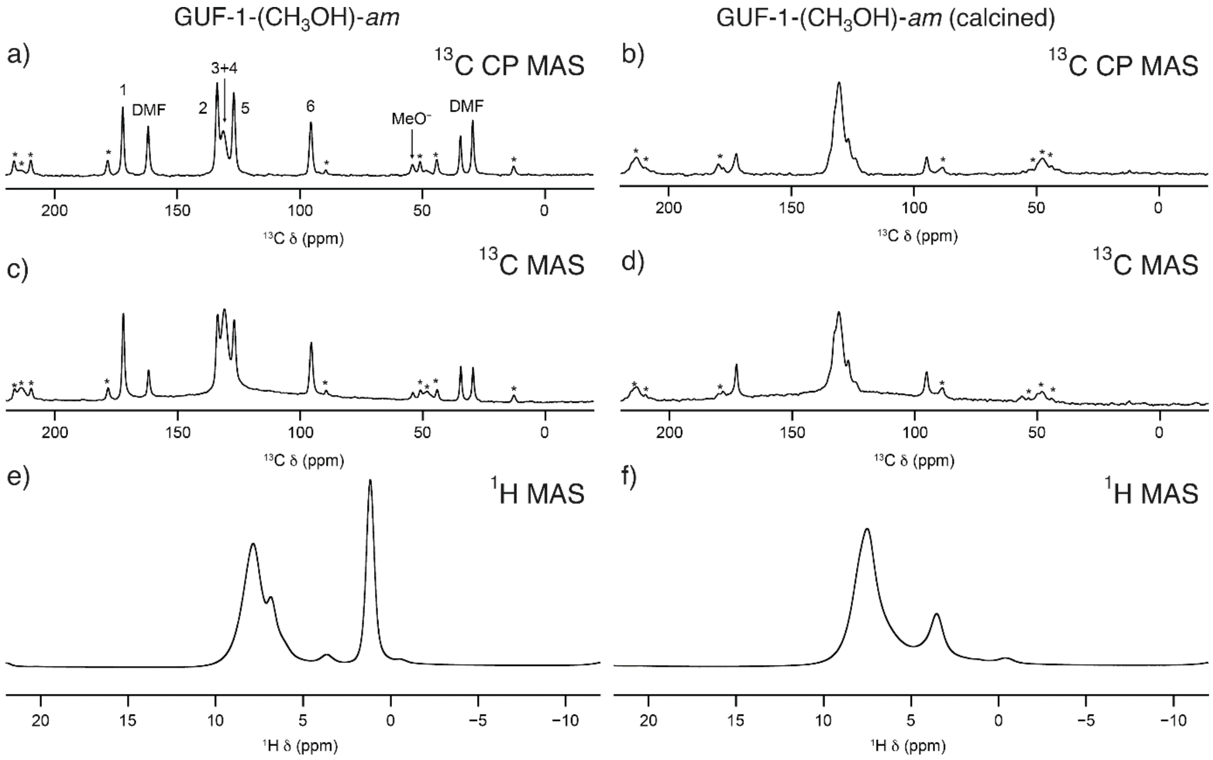

Figure S3. ^{13}C CP (14.1 T, 12.5 kHz) MAS NMR spectra of GUF-1-(CH_3OH)-*am* a) after soaking, and b) after subsequent calcination. ^{13}C (14.1 T, 12.5 kHz) MAS NMR spectra of GUF-1-(CH_3OH)-*am* c) after soaking, and d) after subsequent calcination. ^{1}H (14.1 T, 12.5 kHz) MAS NMR spectra of GUF-1-(CH_3OH)-*am* e) after soaking and f) after subsequent calcination. Asterisks (*) denote spinning sidebands. Numbering scheme for resonances in part a) in Figure S1.

S5.3 GUF-1-($^{13}CH_3OH$)-*am* and GUF-1-($^{13}CH_3OH$)-*P* – Minor Impurity

Samples of GUF-1 were exchanged with $^{13}CH_3OH$ (99% ^{13}C) to amplify the resonance of the μ_2-OCH_3 group in the ^{13}C NMR spectra. ^{13}C CP and ^{1}H NMR spectra are presented in Figures

S4 and S5, respectively, to complement the ^{13}C MAS NMR spectra shown in Figures 3a and 3b in the main text.

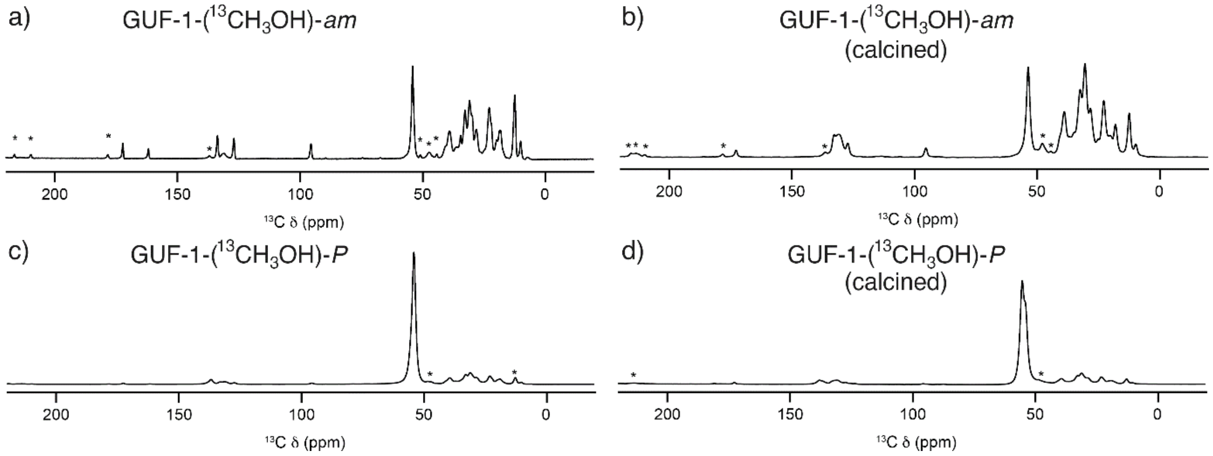

Figure S4. ^{13}C CP (14.1 T, 12.5 kHz) MAS NMR spectra for GUF-1-($^{13}CH_3OH$)-*am* a) after soaking, and b) after subsequent calcination, and for GUF-1-($^{13}CH_3OH$)-*P* c) after soaking, and d) after subsequent calcination.

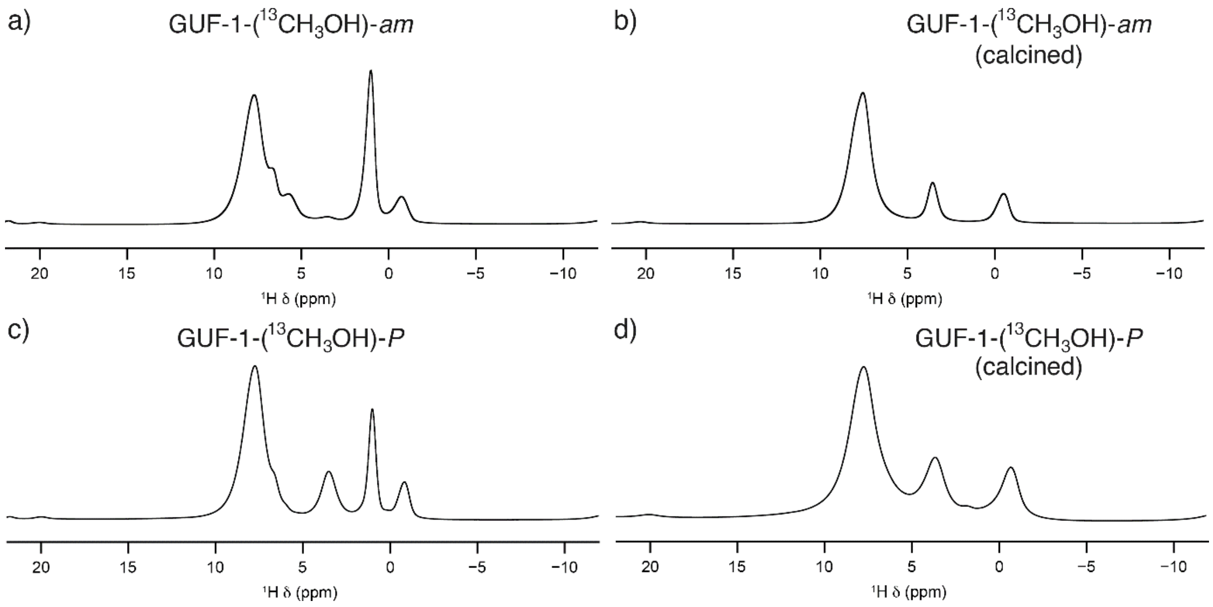

Figure S5. ^{1}H (14.1 T, 12.5 kHz) MAS NMR spectra for GUF-1-($^{13}CH_3OH$)-*am* a) after soaking, and b) after subsequent calcination, and for GUF-1-($^{13}CH_3OH$)-*P* c) after soaking, and d) after subsequent calcination.

The ^{13}C MAS NMR spectra of GUF-1-($^{13}CH_3OH$)-*am* (both post soaking and after calcination) contain unexpected additional resonances between 0 and 50 ppm (Figure 3a in the main text). The large signal intensity when compared to peaks relating to the MOF indicates that the material from which these originate is ^{13}C enriched, *i.e.*, the $^{13}CH_3OH$ in which the MOF was soaked. Their relative intensity does not change following calcination suggesting this impurity is potentially a large molecule or cluster. Assuming 100% ^{13}C enrichment of the impurity, integration indicates it is present as ~2.3% of the total material. These signals are also present in the ^{13}C MAS NMR spectra of GUF-1-($^{13}CH_3OH$)-*P* (Figure 3b in the main text) but have lower relative intensity compared to the resonance corresponding to the μ_2-OCH_3 group due to the greater exchange at high pressure.

The ^{1}H and ^{13}C liquid-state NMR spectra of the $^{13}CH_3OH$ solvent, diluted in D_2O, recovered following the GUF-1-($^{13}CH_3OH$)-*am* soaking procedure are shown in Figure S6.

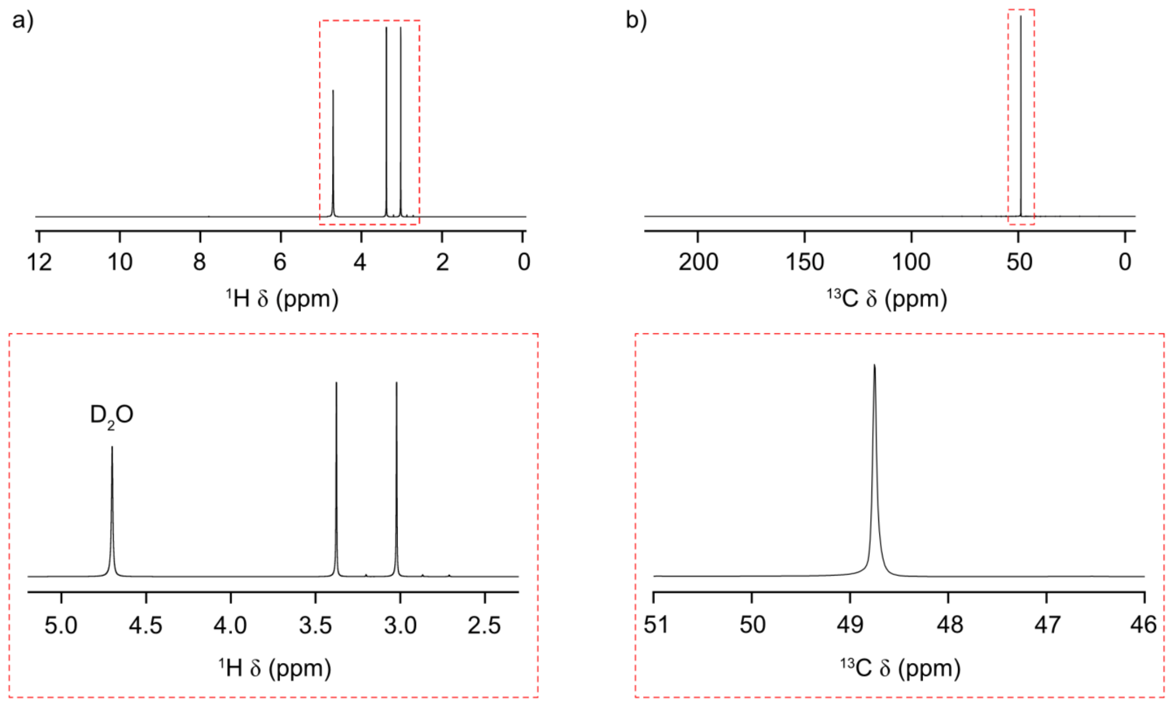

Figure S6. a) ^{1}H and b) ^{13}C DEPTQ (9.4 T) liquid-state NMR spectra of the $^{13}CH_3OH$ solvent, diluted with D_2O, recovered after the GUF-1-($^{13}CH_3OH$)-*am* soaking procedure, showing the presence of $^{13}CH_3OH$ and D_2O. Insets of both spectra are shown within the red-dashed boxes. a) δ_H (400 MHz, D_2O) 3.2 (d, $^{1}J_{HC}$ 144 Hz), 3.2 (s). b) δ_C (100 MHz, D_2O) 48.8 (s).

These NMR spectra show only the presence of $^{13}CH_3OH$, as seen by the presence of only one signal in the ^{13}C NMR spectrum and a doublet at 3.2 ppm in the ^{1}H NMR spectrum, arising from $^{1}J_{HC}$ coupling (144 Hz) between the ^{1}H and ^{13}C nuclei. A small peak is present at 3.2 ppm as a singlet due to the small proportion of $^{12}CH_3OH$ present. An O<u>H</u> signal is not observed in

the ^{1}H NMR spectrum due to ^{2}H exchange between D_2O and CH_3OH. These data suggest the solvent is not the source of the impurity found in GUF-1-($^{13}CH_3OH$) following the soaking step and therefore must either be present in the framework prior to the exchange or result from a reaction between GUF-1 and $^{13}CH_3OH$.

$^{1}H-^{13}C$ HETCOR and ^{13}C INADEQUATE MAS NMR spectra of GUF-1-($^{13}CH_3OH$)-*am* were acquired to gain further insight into the origin of these signals. In the $^{1}H-^{13}C$ HETCOR MAS NMR spectra, Figures S7a and S7b, the majority of ^{13}C NMR signals between 0 and 50 ppm correlate to the ^{1}H signal at −0.5 ppm (indicated by the red lines) whilst some do not appear to correlate with any ^{1}H NMR signals. The centre of these cross peaks varies slightly in δ_1 suggesting that there is more than one ^{1}H environment contributing to this signal. None of the ^{13}C NMR signals corresponding to the organic linker correlate to the ^{1}H resonance at –0.5 ppm, indicating that the impurity is likely to be spatially separate from the MOF framework. As the ^{1}H signal at −0.5 ppm is also present in the ^{1}H MAS NMR spectrum of GUF-1-(DMF)-*am*, it suggests the impurity is present in some level before the exchange process occurs and thus possibly arises during the initial MOF synthesis. As the ^{1}H signal at −0.5 ppm is also present in the ^{1}H MAS NMR spectrum of GUF-1-(DMF)-*am*, it suggests an impurity is present at some level before the exchange process occurs. As discussed earlier, the impurity identified in the ^{13}C NMR spectrum is only present in small quantities (~2%) and therefore it is likely to be observed only when enriched, and would not be identifiable crystallographically. This, however, poses additional questions around how this impurity arises and the role $^{13}CH_3OH$ plays in its development, for which further work would need to be undertaken.

These 2D NMR spectra also enable confirmation of the assignment of the signals in the ^{1}H NMR spectra that relate to DMF, with cross peaks observed in Figure S7a at δ_1 = 6.8 ppm and δ_2 = 161 ppm, and at δ_1 = 1.1 ppm and δ_2 = 35, 30 ppm (shown in green). Two cross peaks are observed for the ^{13}C NMR μ_2-OCH_3 signal (δ_2 = 54 ppm) in Figure S7b, at δ_1 = 3.6 and 7.8 ppm (indicated by the blue lines), with the former indicating the ^{1}H NMR resonance for the methyl group and the latter providing evidence that the methoxy group is bound to the framework. The ^{13}C INADEQUATE MAS NMR spectra, Figures S7c and S7d, show correlations between directly bonded ^{13}C spins, in the form of a pair of cross peaks, situated either side of the central, 2:1 (δ_1: δ_2), diagonal (blue dashed line). These cross-peak pairs are indicated by the red lines in Figure S7d for calcined GUF-1-($^{13}CH_3OH$)-*am*. Analysis of these cross peaks indicates that all the identifiable ^{13}C NMR signals within the region of 0 to 50 ppm correlate with one another, indicating that the impurity is likely to be one large molecule.

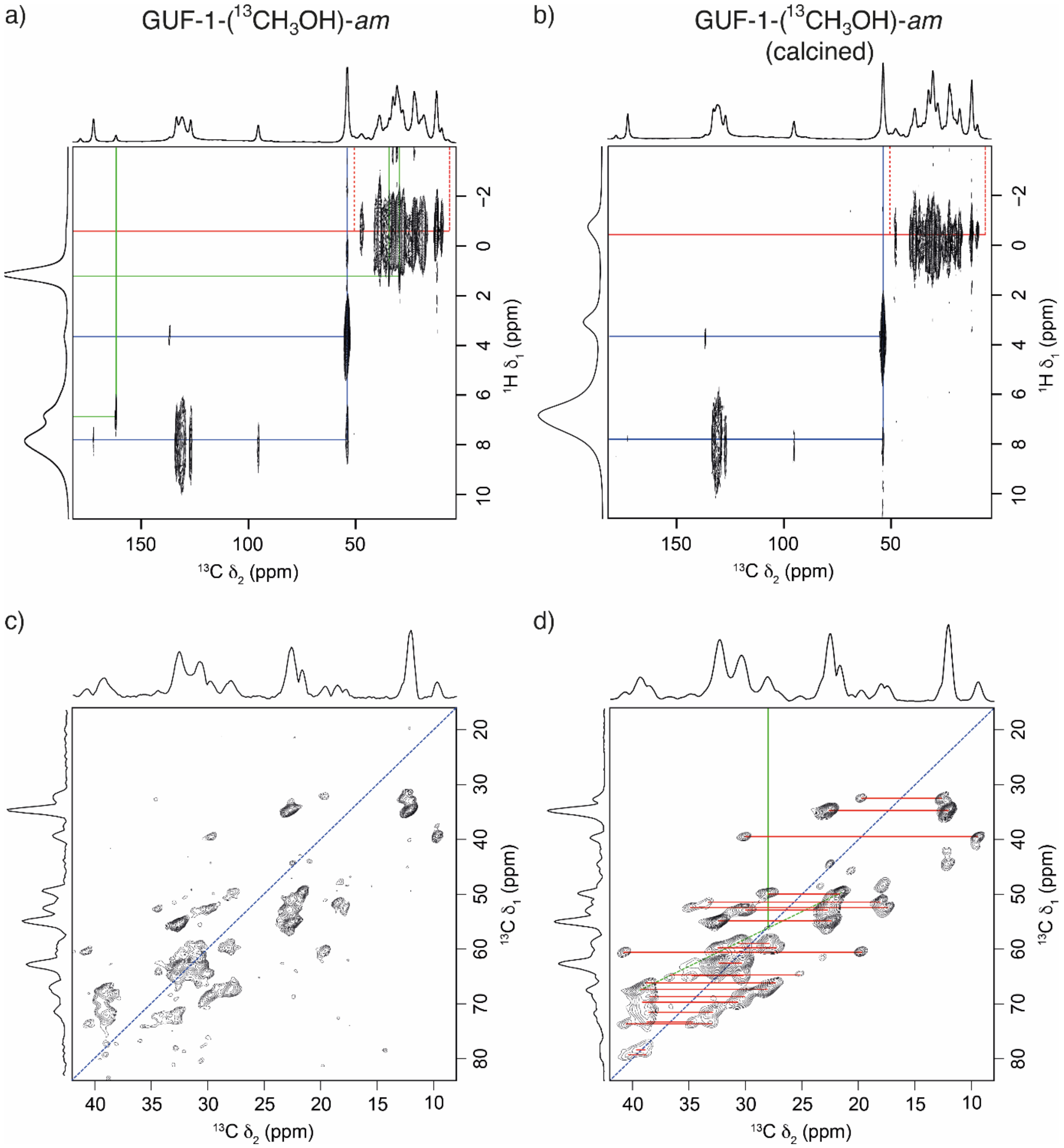

Figure S7. a) and b) $^{1}H-^{13}C$ HETCOR (14.1 T, 12.5 kHz) MAS NMR spectra of GUF-1-($^{13}CH_3OH$)-*am* a) after soaking, and b) after subsequent calcination. ^{13}C INADEQUATE (14.1 T, 12.5 kHz) MAS NMR spectra of GUF-1-($^{13}CH_3OH$)-*am* c) after soaking, and d) after subsequent calcination. The NMR spectra displayed next to the $^{1}H-^{13}C$ HETCOR MAS NMR spectra are the respective ^{1}H and ^{13}C MAS NMR spectra from Figures S4a and S4b, and Figure 3a (main paper), not projections.

To aid assignment, as an example, a 1:1 (δ_1: δ_2) diagonal (green dashed line) is shown which crosses all the peaks which correlate to the ^{13}C NMR signal at δ_2 = 28 ppm. No cross peaks are observed between the impurity and MOF signals, providing additional evidence that the impurity is not bound to the GUF-1 framework. Furthermore, an additional resonance is also observed at −0.5 ppm in the ^{2}H MAS NMR spectra of both GUF-1-(CD_3OD)-*am* and GUF-1-(CD_3OD)-*P* (see Figures 3c and 3d in the main text) that can also be attributed to this minor impurity, which now appears deuterated. The C_Q value associated with this signal is 47(3) kHz (η_Q = 1.0(2)) suggesting that the impurity also has limited rotation. It should be noted that this is an apparent η_Q value of 1.0 due to restricted dynamics (and/or disorder) and not a true measurement of the asymmetry of a single quadrupolar tensor. This corroborates the data discussed above, indicating the impurity is likely to be a single large molecule trapped within the porosity of the MOF.

S6. Scale-Up and Bulk Characterisation

S6.1. GUF-1-(AcOH) Control

In our previous reports of GUF-1, we showed that porosity could be optimised by a modulated synthesis approach using both L-proline and AcOH as co-modulators.[S5] Our investigation of the GUF-1 methanol substitution phenomenon pre-dated this porosity optimisation, and so bulk scale samples of the MOF for analysis by solid-state NMR spectroscopy were prepared by conventional AcOH modulation. GUF-1-(AcOH) was prepared by the method in Section 2.3 and characterised by powder X-ray diffraction analysis and nitrogen adsorption/desorption isotherms. To determine the effect of methanol washing, a benchmark sample of GUF-1-(AcOH) was prepared and activated by washing three times with MeOH (5 ml, 10 min) followed by heating to 150 °C under vacuum (turbo pump) for 20 h. The powder X-ray diffractogram of the sample before activation (Figures S8 and S9) is typical of the samples that were analysed using solid-state NMR spectroscopy. After activation, some minor structural changes have occurred which may reflect the limited flexibility of the GUF-1 framework.

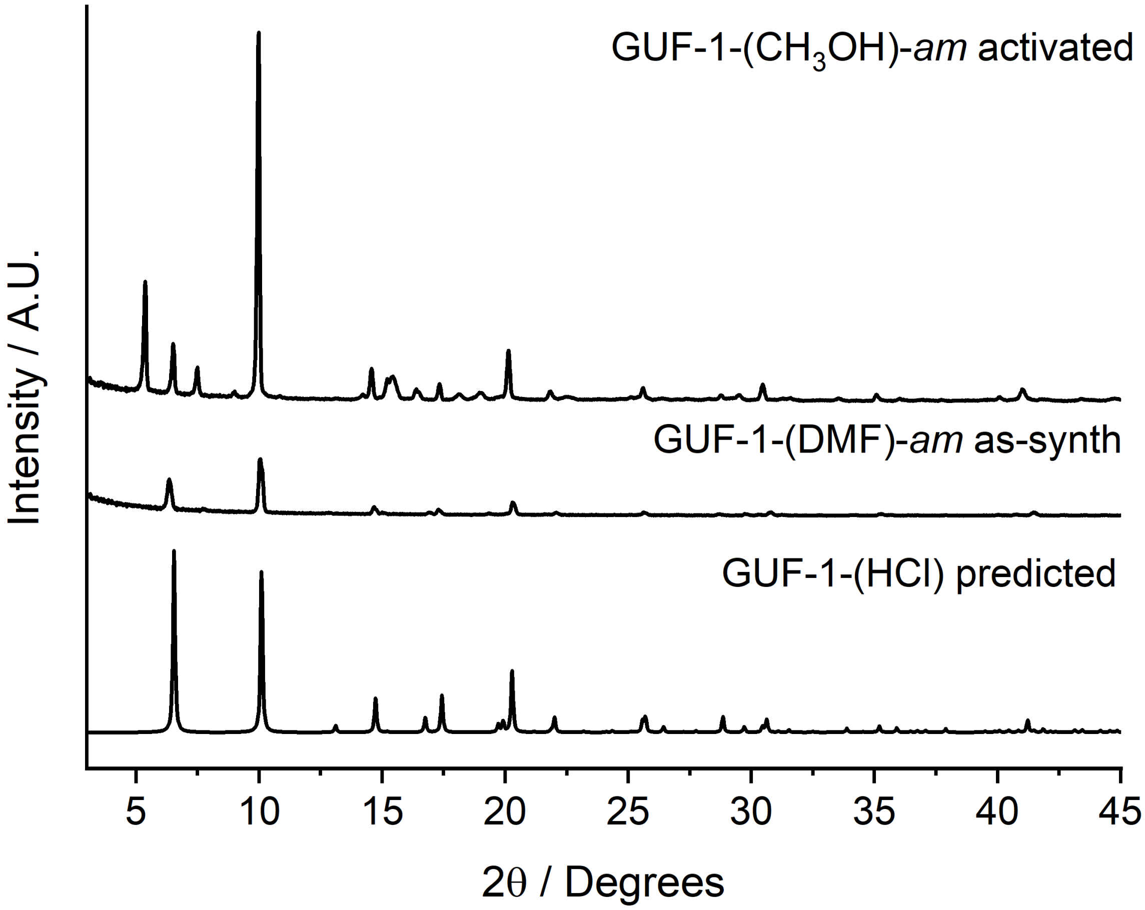

Figure S8. Diffractograms of GUF-1-(DMF)-*am* before and after soaking in CH_3OH and subsequent activation compared to the predicted pattern of GUF-1-(HCl).

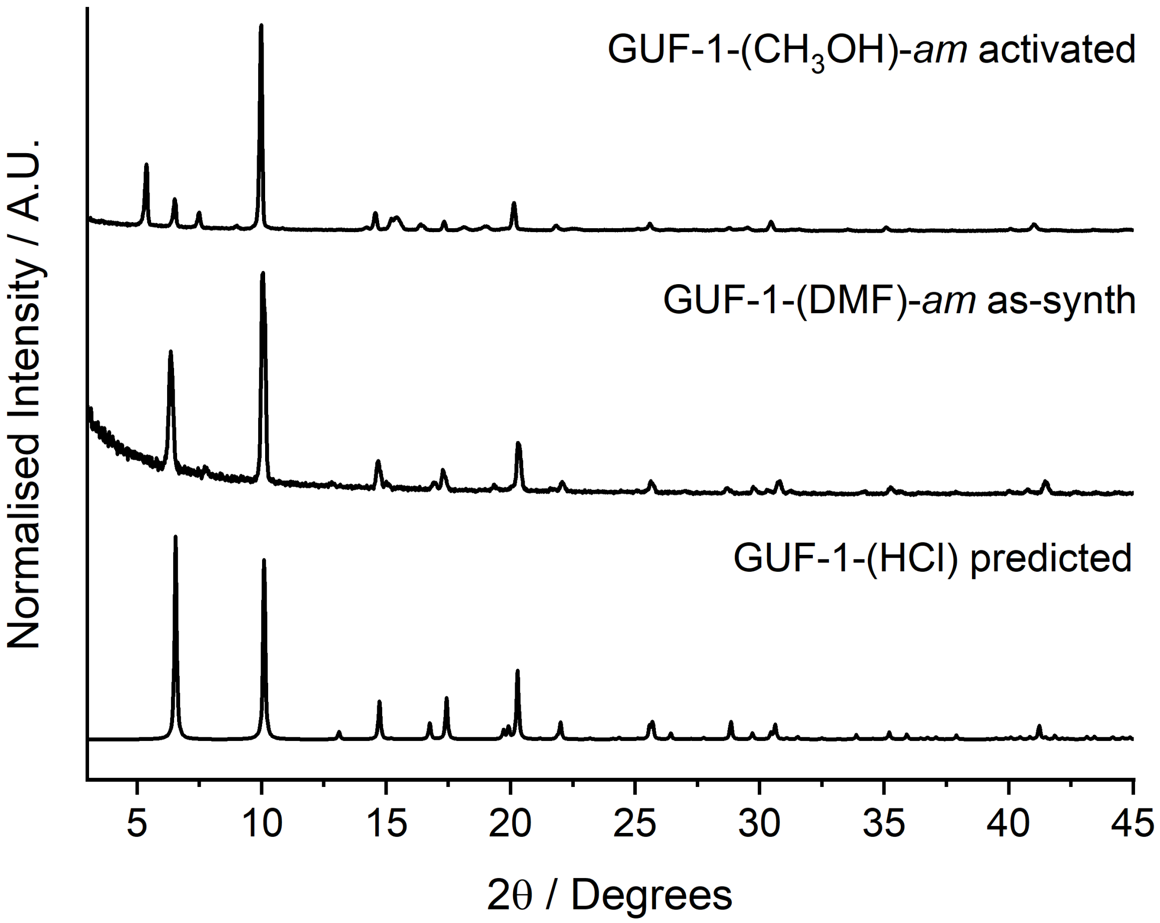

Figure S9. Diffractograms of GUF-1-(DMF)-*am* before and after soaking in CH_3OH and subsequent activation compared to the predicted pattern of GUF-1-(HCl) with normalised intensities.

Porosity measurements (Figures S10–S12) showed that this sample of GUF-1-(CH_3OH)-*am* had a BET area (calculated using the BETSI[S20] software package) of 688 m^2 g^{-1} and a total N_2 uptake of 245 cc g^{-1} at P/P_0 = 1, while the isotherm retained the characteristic step at P/P_0 ~0.1, indicative of flexibility, and hysteresis in the desorption profile. The uptake is higher than corresponding acetone activated GUF-1-(AcOH), but still lower than acetone activated GUF-1-(L-Pro/AcOH).[S5] Nevertheless, GUF-1-(CH_3OH)-*am* retains porosity and crystallinity.

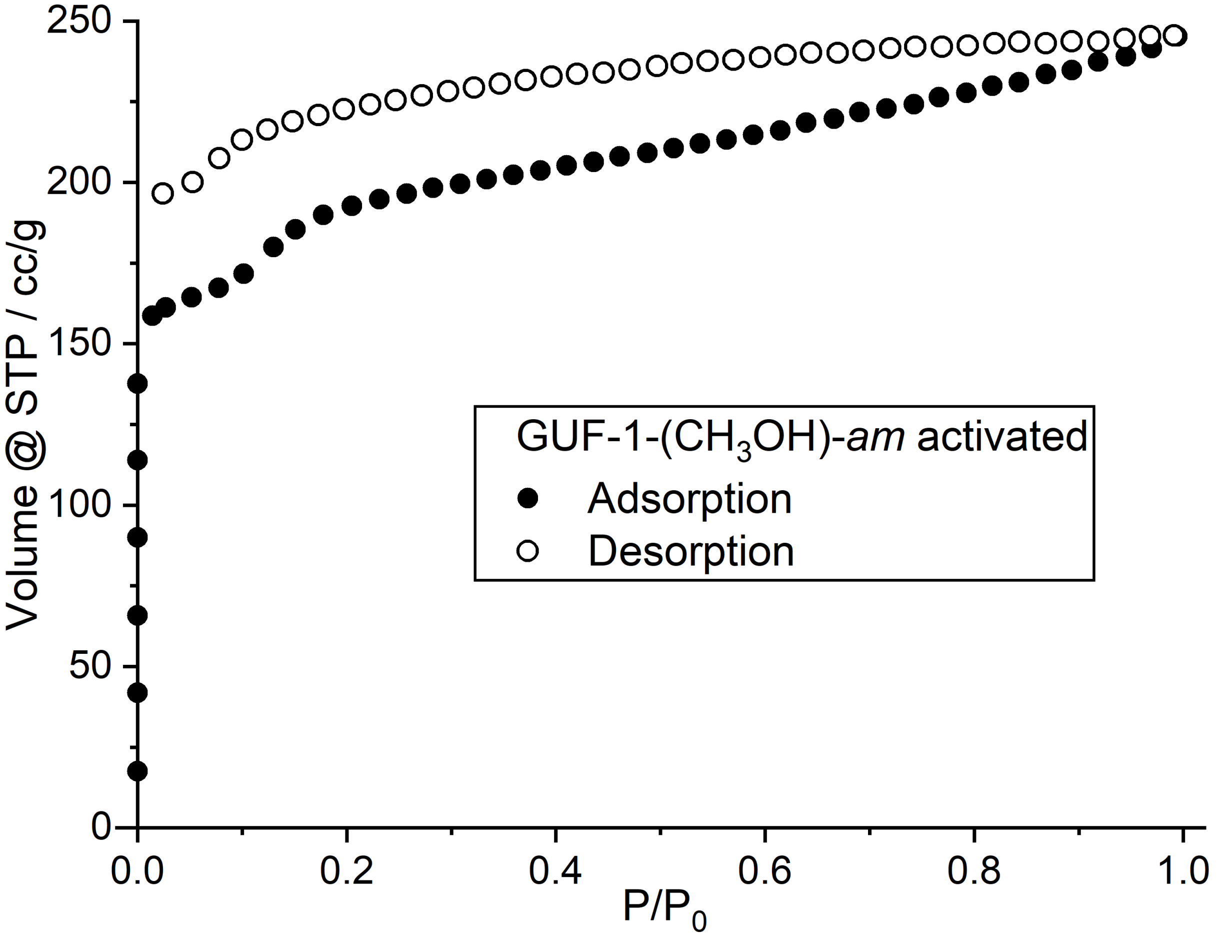

Figure S10. N_2 adsorption/desorption isotherm (77 K) GUF-1-(CH_3OH)-*am* after activation.

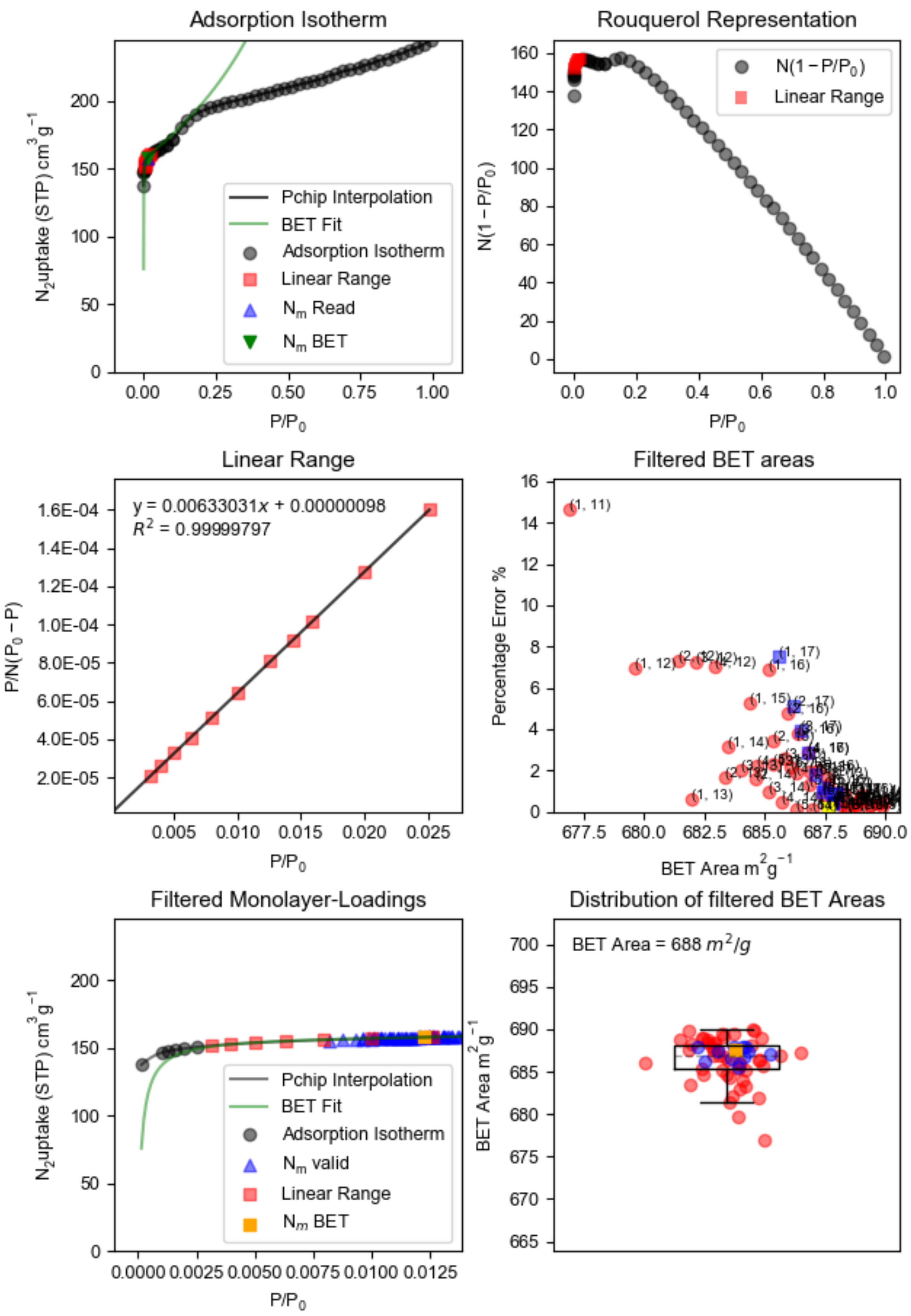

Figure S11. BETSI analysis for GUF-1-(CH_3OH)-*am* derived from the N_2 adsorption/desorption isotherm (Figure S10).

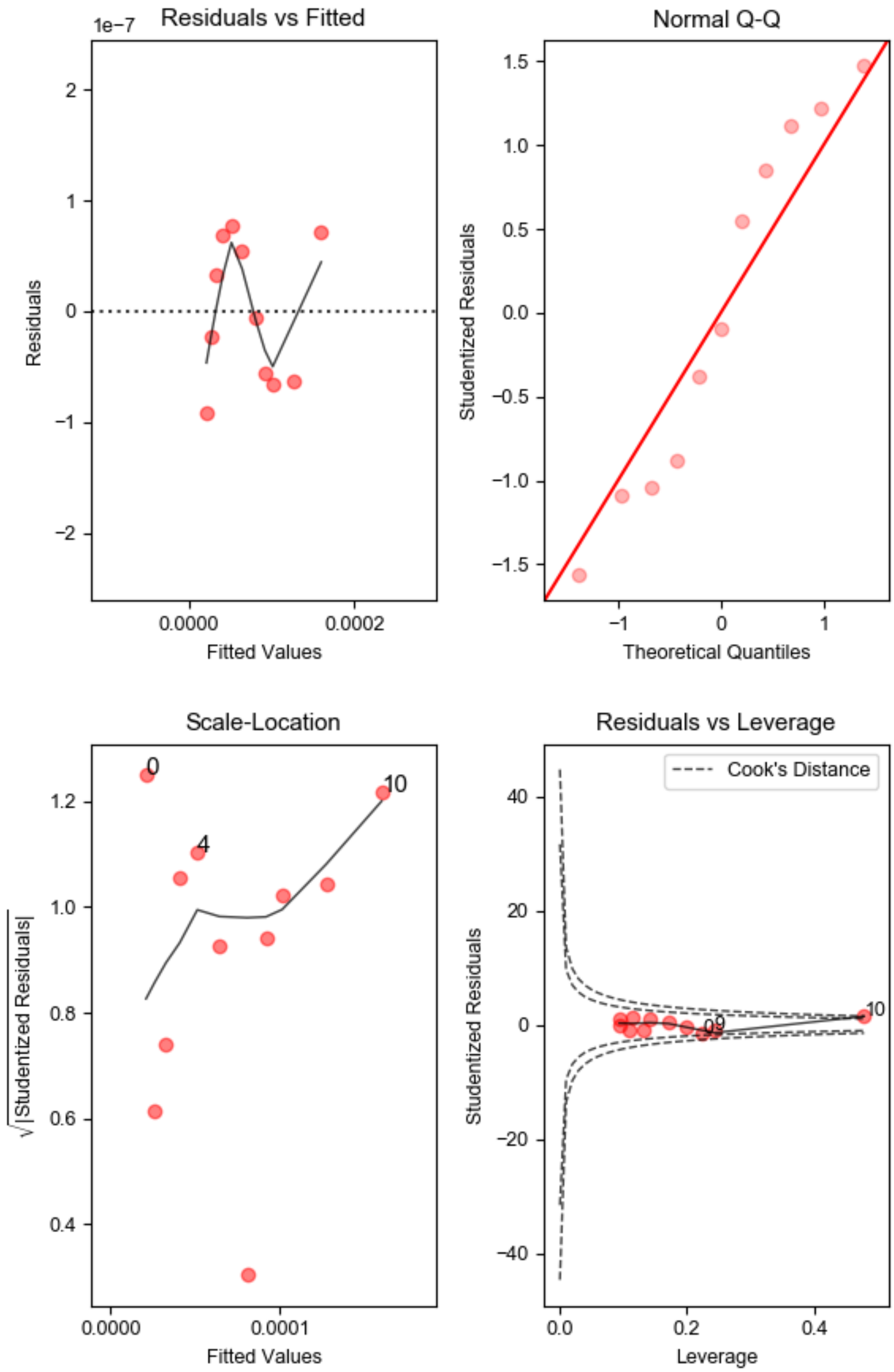

Figure S12. BETSI regression diagnostics for GUF-1-(CH_3OH)-*am* derived from the N_2 adsorption/desorption isotherm (Figure S10).

After completion of the comprehensive solid-state NMR spectroscopic analysis of methanol substitution of GUF-1-(AcOH), analogous samples to GUF-1-(CH_3OH)-*am* and GUF-1-(CH_3OH)-*P* were prepared by activating our previously reported[S5] GUF-1-(L-Pro/AcOH) in CH_3OH. These materials were analysed using the same ^{13}C and ^{1}H solid-state NMR experiments to allow comparisons of the bulk material to those compounds activated from GUF-1-(AcOH), with the spectra acquired after calcination shown in Figure S13. Comparison of these spectra show the same resonances are present and the same level of μ_2-OCH_3 substitution, confirming that GUF-1-(AcOH) is a valid material for testing this phenomenon in bulk.

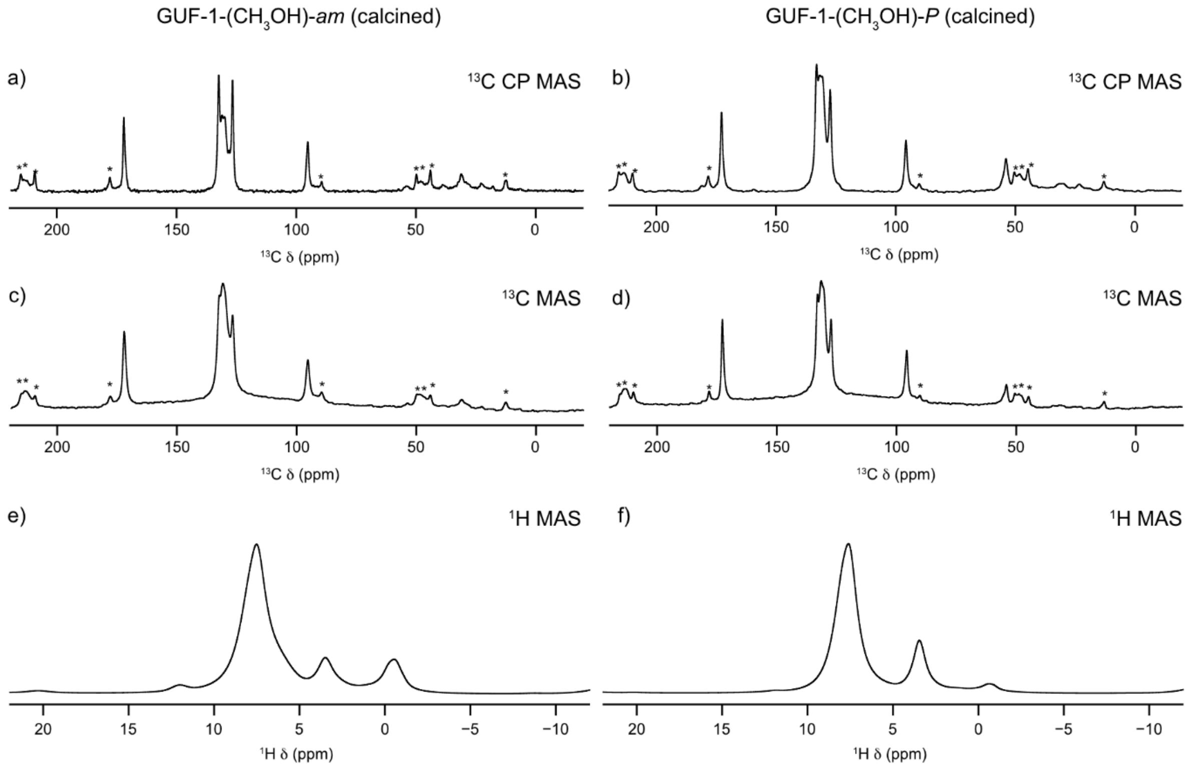

Figure S13. Analysis of analogous GUF-1-(CH_3OH) materials prepared from GUF-1-(L-Pro/AcOH). ^{13}C CP (14.1 T, 12.5 kHz) MAS NMR spectra of a) GUF-1-(CH_3OH)-*am* and b) GUF-1-(CH_3OH)-*P* after calcination. ^{13}C (14.1 T, 12.5 kHz) MAS NMR spectra of c) GUF-1-(CH_3OH)-*am* and d) GUF-1-(CH_3OH)-*P* after calcination. ^{1}H (14.1 T, 12.5 kHz) MAS NMR spectra of e) GUF-1-(CH_3OH)-*am* and f) GUF-1-(CH_3OH)-*P* after calcination. Asterisks (*) denote spinning sidebands.

S6.2. Analysis of Samples used in Solid-State NMR Measurements

Powder X-ray diffraction analysis was used to probe the integrity of the bulk samples of GUF-1-(AcOH) that were studied by solid-state NMR spectroscopy. Samples were analysed after bulk pressurisation at 0.7 GPa and subsequently after calcination. These data were compared with the diffractograms of the analogous materials soaked at ambient pressure after calcination (Figures S14–S16). As with the control sample (Figures S8 and S9) it is the calcination/activation process, rather than the bulk pressurisation, that induces minor structural variations across the samples. Comparison of the calcined samples that were reacted with the various methanol isotopologues under ambient conditions with those reacted under pressure shows that the materials are very similar, and that pressurisation has not induced any significant structural change.

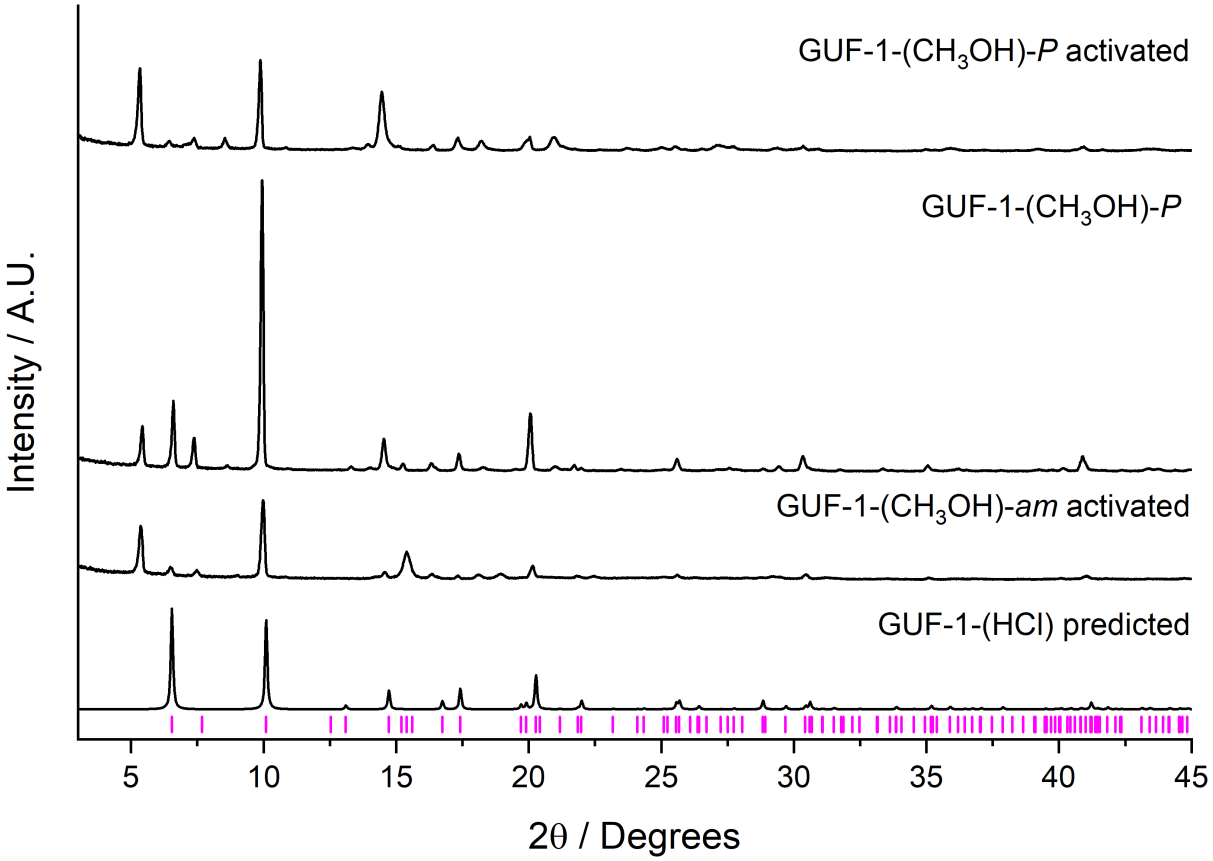

Figure S14. Stacked powder X-ray diffractograms of GUF-1 samples after treatment with CH_3OH and subsequent activation ahead of solid-state NMR spectroscopic experiments.

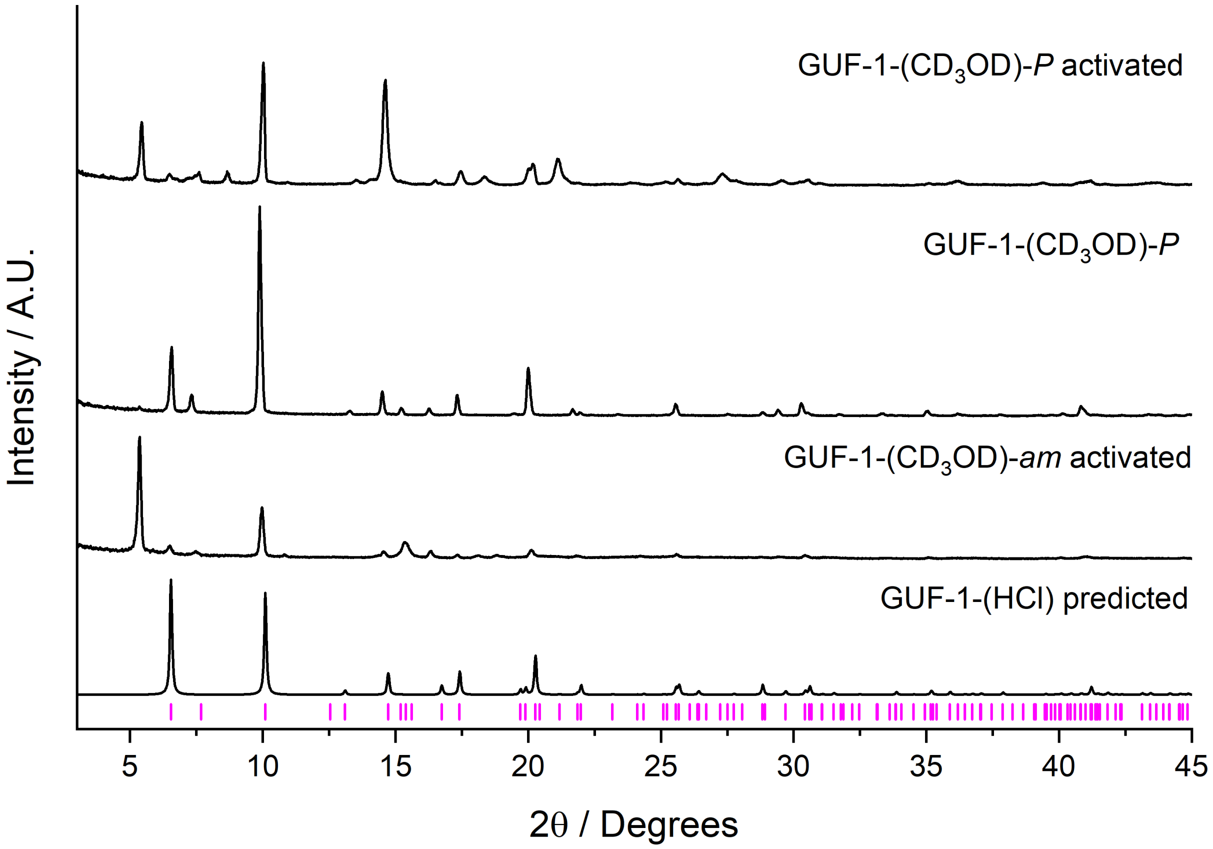

Figure S15. Stacked powder X-ray diffractograms of GUF-1 samples after treatment with CD_3OD and subsequent activation ahead of solid-state NMR spectroscopic experiments.

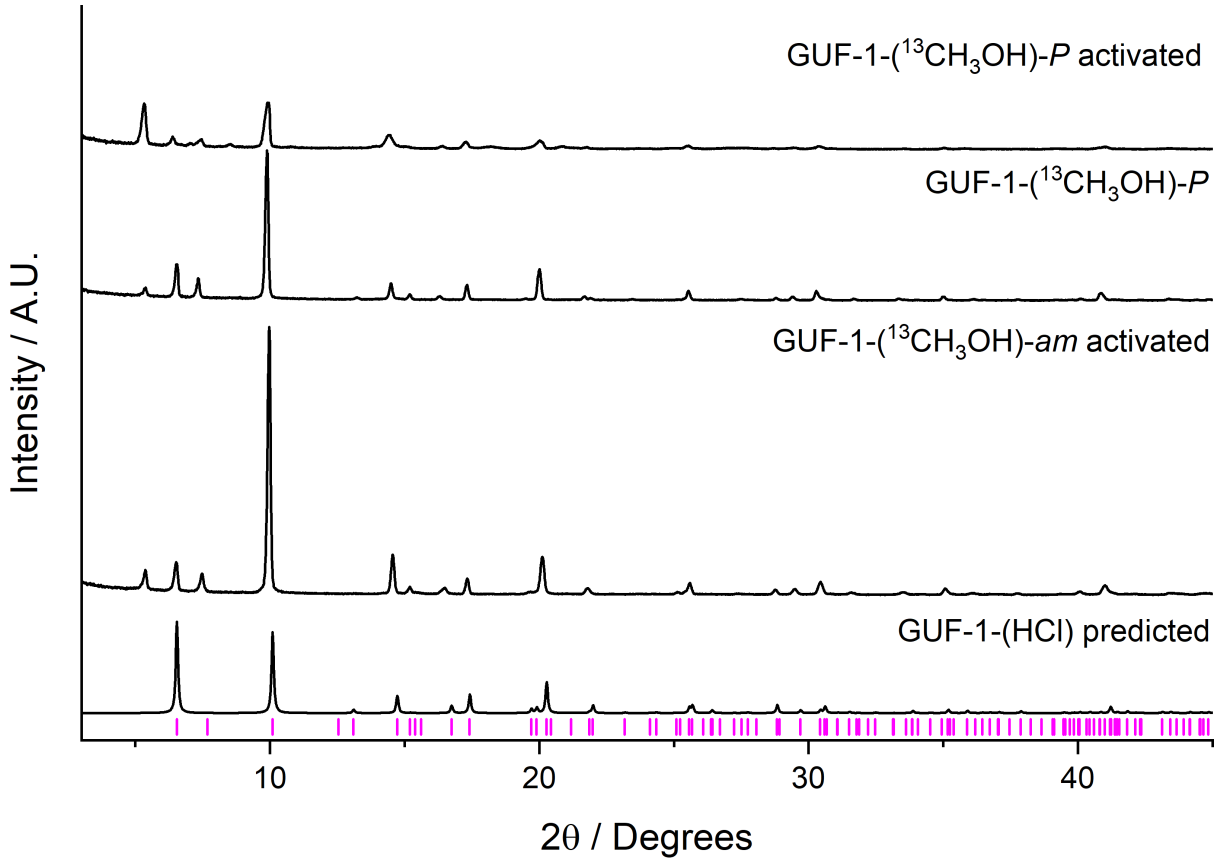

Figure S16. Stacked powder X-ray diffractograms of GUF-1 samples after treatment with $^{13}CH_3OH$ and subsequent activation ahead of solid-state NMR spectroscopic experiments.

S6.3. GUF-1-(CH_3OH)-*reflux*

To compare the effects of temperature and pressure on the cluster anion substitution process, a sample of GUF-1-(AcOH) was refluxed in natural abundance CH_3OH for 16 h, which was termed GUF-1-(CH_3OH)-*reflux*, and subsequently analysed by solid state NMR spectroscopy (Figure S17). Integrating the quantitative ^{13}C MAS NMR spectra of GUF-1-(CH_3OH)-*reflux* quantifies the exchange for μ_2-OCH_3 as 52(2)%, which decreases to 46(2)% upon calcination of the sample at 140 °C for 48 hours under a reduced pressure of 10^{-4} Torr, suggesting the presence of a small amount of free CH_3OH within the framework pores after the initial high temperature solvent exchange and drying. We note that there is background signal present in the quantitative ^{13}C MAS NMR spectra which has been accounted for when extracting information on the relative proportions of each signal to determine the level of μ_2-OCH_3 exchange. This background signal arises from the use of a alternative 4 mm HX MAS NMR probe for these particular experiments. Despite this, the results obtained here are still

comparable to other NMR spectra presented in this work. As seen for the other GUF-1-(CH_3OH) materials, there is again a small impurity peak present in the ^{1}H MAS NMR spectra at –0.5 ppm. Likewise, an additional resonance is present in the ^{13}C MAS NMR spectra at ~31 ppm, which like also relates to this impurity. The smaller intensity of this signal likely results from the natural abundance CH_3OH used in the preparation of this material.

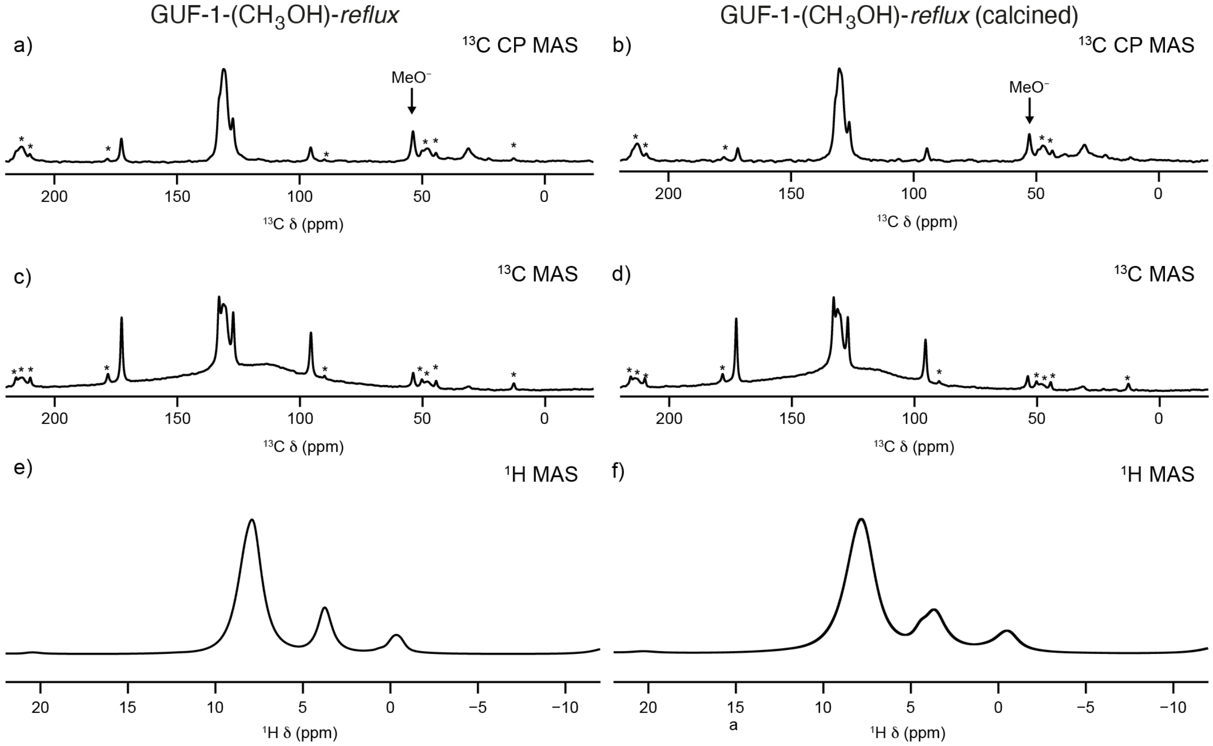

Figure S17. Solid-state NMR spectroscopic analysis of GUF-1-(CH_3OH)-*reflux*. ^{13}C CP (14.1 T, 12.5 kHz) MAS NMR spectra of a) GUF-1-(CH_3OH)-*reflux* and b) GUF-1-(CH_3OH)-*reflux* after calcination. ^{13}C (14.1 T, 12.5 kHz) MAS NMR spectra of c) GUF-1-(CH_3OH)-*reflux* and d) GUF-1-(CH_3OH)-*reflux* after calcination. ^{1}H (14.1 T, 12.5 kHz) MAS NMR spectra of e) GUF-1-(CH_3OH)-*reflux* and f) GUF-1-(CH_3OH)-*reflux* after calcination. Asterisks (*) denote spinning sidebands.

S7. References

S1. I. B. Hutchison, A. Delori, X. Wang, K. V. Kamenev, A. J. Urquhart and I. D. H. Oswald, *CrystEngComm*, 2015, **17**, 1778-1782.

S2. A. Pines, M. G. Gibby and J. S. Waugh, *J. Chem. Phys.*, 1972, **56**, 1776-1777.

S3. A. E. Bennett, C. M. Rienstra, M. Auger, K. V. Lakshmi and R. G. Griffin, *J. Chem. Phys.*, 1995, **103**, 6951-6958.

S4. A. Lesage, M. Bardet and L. Emsley, *J. Am. Chem. Soc.*, 1999, **121**, 10987-10993.

S5. A. J. R. Thom, D. G. Madden, R. Bueno-Perez, A. N. Al Shakhs, C. T. Lennon, R. J. Marshall, C. A. Walshe, C. Wilson, C. A. Murray, S. P. Thompson, G. F. Turner, D. Bara, S. A. Moggach, D. Fairen-Jimenez and R. S. Forgan, *Mater. Today Chem.*, 2022, **24**, 100887.

S6. APEX3 Package, APEX3, SAINT and SADABS, **2016**. Bruker Corporation, USA.

S7. G. Sheldrick, *Acta Cryst. A*, 2015, **71**, 3-8.

S8. G. Sheldrick, *Acta Cryst. C*, 2015, **71**, 3-8.

S9. O. V. Dolomanov, L. J. Bourhis, R. J. Gildea, J. A. K. Howard and H. Puschmann, *J. Appl. Crystallogr.*, 2009, **42**, 339-341.

S10. L. Merrill and W. A. Bassett, *Rev.Sci. Instrum.*, 1974, **45**, 290-294.

S11. D. R. Allan, S. J. Clark, M. J. P. Brugmans, G. J. Ackland and W. L. Vos, *Phys. Rev. B*, 1998, **58**, R11809–R11812.

S12. G. J. Piermarini, S. Block, J. D. Barnett and R. A. Forman, *J. Appl. Phys.*, 1975, **46**, 2774-2780.

S13. A. Dawson, D. R. Allan, S. Parsons and M. Ruf, *J. Appl. Crystallogr.*, 2004, **37**, 410-416.

S14. CrysAlisPro, **2017**. Rigaku Corporation, Oxford, UK.

S15. SADABS, **1996**. G. Sheldrick, University of Göttingen, Germany.

S16. A. Spek, *Acta Cryst. C*, 2015, **71**, 9-18.

S17. A. Spek, *J. Appl. Crystallogr.*, 2003, **36**, 7-13.

S18. C. F. Macrae, I. Sovago, S. J. Cottrell, P. T. A. Galek, P. McCabe, E. Pidcock, M. Platings, G. P. Shields, J. S. Stevens, M. Towler and P. A. Wood, *J. Appl. Crystallogr.*, 2020, **53**, 226-235.

S19. J. P. S. Mowat, S. R. Miller, A. M. Z. Slawin, V. R. Seymour, S. E. Ashbrook and P. A. Wright, *Microporous Mesoporous Mater.*, 2011, **142**, 322-333.

S20. J. W. M. Osterrieth, J. Rampersad, D. Madden, N. Rampal, L. Skoric, B. Connolly, M. D. Allendorf, V. Stavila, J. L. Snider, R. Ameloot, J. Marreiros, C. Ania, D. Azevedo, E.

Vilarrasa-Garcia, B. F. Santos, X.-H. Bu, Z. Chang, H. Bunzen, N. R. Champness, S. L. Griffin, B. Chen, R.-B. Lin, B. Coasne, S. Cohen, J. C. Moreton, Y. J. Colón, L. Chen, R. Clowes, F.-X. Coudert, Y. Cui, B. Hou, D. M. D'Alessandro, P. W. Doheny, M. Dincă, C. Sun, C. Doonan, M. T. Huxley, J. D. Evans, P. Falcaro, R. Ricco, O. Farha, K. B. Idrees, T. Islamoglu, P. Feng, H. Yang, R. S. Forgan, D. Bara, S. Furukawa, E. Sanchez, J. Gascon, S. Telalović, S. K. Ghosh, S. Mukherjee, M. R. Hill, M. M. Sadiq, P. Horcajada, P. Salcedo-Abraira, K. Kaneko, R. Kukobat, J. Kenvin, S. Keskin, S. Kitagawa, K.-i. Otake, R. P. Lively, S. J. A. DeWitt, P. Llewellyn, B. V. Lotsch, S. T. Emmerling, A. M. Pütz, C. Martí-Gastaldo, N. M. Padial, J. García-Martínez, N. Linares, D. Maspoch, J. A. Suárez del Pino, P. Moghadam, R. Oktavian, R. E. Morris, P. S. Wheatley, J. Navarro, C. Petit, D. Danaci, M. J. Rosseinsky, A. P. Katsoulidis, M. Schröder, X. Han, S. Yang, C. Serre, G. Mouchaham, D. S. Sholl, R. Thyagarajan, D. Siderius, R. Q. Snurr, R. B. Goncalves, S. Telfer, S. J. Lee, V. P. Ting, J. L. Rowlandson, T. Uemura, T. Iiyuka, M. A. van der Veen, D. Rega, V. Van Speybroeck, S. M. J. Rogge, A. Lamaire, K. S. Walton, L. W. Bingel, S. Wuttke, J. Andreo, O. Yaghi, B. Zhang, C. T. Yavuz, T. S. Nguyen, F. Zamora, C. Montoro, H. Zhou, A. Kirchon and D. Fairen-Jimenez, *Adv. Mater.*, 2022, **34**, 2201502.
